# Deciphering the role of Hat1 in spermatogenesis: Chromatin organization and beyond

**DOI:** 10.7717/peerj.20240

**Published:** 2025-11-19

**Authors:** Shenni Peng, Yulian Tang, Ruiqun Lu, Shi Huang, Yinyin Mo, Hailing Huang, Genliang Li

**Affiliations:** Youjiang Medical University for Nationalities, Baise, Guangxi, China

**Keywords:** Hat1, Hat1-bound-protein genes, Chromatin organization, Spermatogenesis, Epigenetic regulation, Mouse testes

## Abstract

Spermatogenesis, a core process for male fertility, relies heavily on chromatin organization regulated by histone acetyltransferases (HATs). However, the spatiotemporal expression pattern of histone acetyltransferase 1 (Hat1) in mouse testes and its specific role in spermatogenesis *via* chromatin organization remain unclear. This study employed RT-qPCR, Western blot, immunofluorescence localization, and bioinformatics to explore Hat1’s dynamic expression and regulatory mechanisms during mouse spermatogenesis. Results showed that both Hat1 mRNA and protein were significantly upregulated in the testes of 8-week-old (mature) mice compared to 3-week-old (immature) mice. Immunofluorescence revealed Hat1 was predominantly localized in the nuclei of male germ cells, with stage-specific expression: highest in spermatogonia and sperm, intermediate in primary spermatocytes, and lowest in secondary spermatocytes. Bioinformatics analysis (based on single-cell sequencing data GSE214315) identified 246 differentially expressed genes (DEGs) related to chromatin organization—these DEGs were screened between adjacent stages of male germ cell development during spermatogenesis, including comparisons of leptotene-zygotene *vs.* pachytene-diplotene cells, pachytene-diplotene *vs.* round spermatids, round spermatids *vs.* early elongating spermatids, and early elongating *vs.* late elongating spermatids (screening criteria: FDR < 0.05, |log2(FC)| ≥ 1). Additionally, 41 Hat1-interacting proteins encoded by these DEGs were identified. Functional enrichment indicated stage-specific roles of Hat1: in the leptotene-zygotene phase, it participated in transcription regulation to initiate meiosis; in round spermatids, it shifted to refined epigenetic regulation and chromatin assembly for subsequent spermiogenesis; in late spermiogenesis and sperm, it was involved in DNA repair and ATP-dependent chromatin remodeling to protect sperm genetic material. In summary, the stage-specific expression patterns of Hat1 and its interactors highlighted the importance of precise control of gene expression and chromatin remodeling, as well as DNA repair in protection of sperm genetic material, in the development of male germ cells. However, future research should conduct functional assays. Overall, this research provides valuable insights into the epigenetic regulatory mechanisms of spermatogenesis and a foundation for male fertility research.

## Introduction

Spermatogenesis, the complex process by which male germ cells develop into mature sperm, is an essential biological phenomenon necessary for the continuation of a species. This complex process involves a series of highly regulated steps, and at its core lies the dynamic organization of chromatin. Chromatin organization in spermatogenesis is a fundamental process that plays a pivotal role in regulating gene expression and ensuring the proper development and maturation of sperm.

Biological processes (BPs) are essential for the proper functioning and maintenance of cells, and spermatogenesis is no exception. The complex web of male fertility is complicatedly woven through the multifaceted process of spermatogenesis, which orchestrates numerous cellular mechanisms (Barbero et al., 2023; Bellil et al., 2021). At the heart of these BPs lies the fundamental cellular process of chromatin organization. Chromatin, a complex structure composed of DNA intricately intertwined with histones, associated proteins, and sometimes RNA, influences the patterns of gene expression and the condensation of chromatin. Both of these aspects are paramount for the development and functionality of male germ cells. Therefore, changes in chromatin organization within cells are indispensable in determining cell fate, including the maintenance of stem cell properties, the initiation and directionality of cell differentiation (Wu et al., 2022; Wang et al., 2019).

Among the regulatory mechanisms of chromatin organization, histone modifications play a significant role (Liu et al., 2025; Oishi et al., 2023). Histone acetyltransferases (Hats) are enzymes that catalyze the addition of acetyl groups to histones, leading to alterations in chromatin structure and gene expression pattern. Histone acetyltransferase 1 (Hat1) is one of the Hats and is known to modulate chromatin conformation and function through histone acetylation (Ortega et al., 2023; Xu et al., 2023; Nagamori, Cruickshank & Sassone-Corsi, 2011). Its aberrant expression and activity are often associated with various cancers and are frequently upregulated and correlated with a poor prognosis (Capone et al., 2023; Klett-Mingo et al., 2022). However, Hat1’s role extends beyond histone acetylation. It also has succinyl transferase activity, targeting histone H3 at lysine 122, and is linked to epigenetic regulation and gene expression in cancer cells (Yang et al., 2021). Moreover, Hat1 can succinylate non-histone proteins, such as the glycolytic enzyme PGAM1, enhancing its activity and promoting cancer cell glycolysis and tumor growth (Wang et al., 2023).

In the context of spermatogenesis, Hat1 is hypothesized to participate in the regulation of spermatid metamorphosis and chromatin condensation, thus playing a potential role in sperm maturation (Liu et al., 2025; Nagamori, Cruickshank & Sassone-Corsi, 2011). The involvement of Hat1 in the process of spermatogenesis in animals is not limited to histone acetylation but also extends to the non-condensation characteristics of the sperm nucleus and chromatin remodeling during sperm maturation. In Chinese mitten crab (*Eriocheir sinensis*), Hat1 expression is higher in mature testes compared to juveniles (Liu et al., 2025). Studies in yaks have also demonstrated differential expression of Hat1 in testis tissue, further indicating a role in spermatogenesis (Sun et al., 2022). Although the intricacies of spermatogenesis can differ significantly among species, the condensation and subsequent decondensation of sperm nuclear genetic material are common stages in the spermatogenesis process. Therefore, it is conceivable that Hat1 could play a similar role in the regulation of spermatid metamorphosis and chromatin condensation across different species.

However, the specific expression patterns and functions of Hat1 in mouse testes, as well as the detailed mechanisms by which Hat1 influences spermatogenesis through chromatin organization, are areas that still require further exploration and clarification. Previous studies have provided some insights into the role of Hat1 (Ortega et al., 2023), but the expression pattern and localization of Hat1 and the evidence linking Hat1 to specific chromatin remodeling events during the mouse spermatogenesis have not been fully characterized.

To address these gaps in knowledge, this study aims to decipher the role of Hat1 in the mouse spermatogenesis with a specific focus on chromatin organization. We hypothesize that Hat1 plays a crucial role in regulating chromatin structure and gene expression at the different stages of spermatogenesis. By using a combination of molecular and bioinformatics techniques, such as reverse transcription quantitative polymerase chain reaction (RT-qPCR), western blot (WB), and immunofluorescence (IF) localization, and analysis of GO (gene ontology) terms, KEGG pathway, and protein-protein interaction (PPI), we will evaluate the expression and localization of Hat1 in mouse testes and investigate its potential functions and interaction with chromatin organization genes. This research has the potential to provide new insights into the complex mechanisms of spermatogenesis and may have important implications for understanding male infertility.

## Materials and Methods

### Animal and tissue collection

Thirty healthy male mice (3 weeks old: 12.5 ± 0.5 g; 8 weeks old: 27.5 ± 2.5 g) were purchased from Changsha Tianqin Biotechnology Co., Ltd., with five mice per experimental group (*n* = 5, randomized). Mice were housed in a specific pathogen-free (SPF) facility at 22 ± 2 °C, 50 ± 5% relative humidity, and a 12-h light-dark cycle, with ad libitum access to standard chow and water, and environmental enrichment (nesting material, small toys). After 1–2 days of acclimation, mice were anesthetized *via* intraperitoneal injection of ketamine (75 mg/kg) + dexmedetomidine (0.5 mg/kg); euthanasia was performed by cervical dislocation when deep, stable anesthesia (no response to tail/paw pinching) was achieved. Testis tissues were isolated, pooled (5 mice/group), and divided for immediate use or storage at −20 °C/−80 °C.

This study complied with ARRIVE 2.0 guidelines and was approved by the Experimental Animal Ethics Committee of Youjiang Medical College for Nationalities (approval no. 2024032901; SYXK(Gui)2017-0004). All data are available from the Supplemental Files; sequencing data are from the GEO repository (https://www.ncbi.nlm.nih.gov/geo/query/acc.cgi?acc=GSE214315). No animals or data points were excluded.

### RT-qPCR analysis of Hat1 expression in mouse testes

Total RNA was extracted from 200 mg testis tissue (3/8-week-old mice) using a Total RNA Extraction Kit (Solabao, Beijing, China). RNA purity/concentration was determined by A260/280 and A260/230 ratios (ultra-micro spectrophotometer). cDNA was synthesized with the BeyoRT cDNA Synthesis Kit (Beyotime, Shanghai, China; including gDNA removal) using a Gradient PCR System (Tiangen, Beijing, China), and stored at −20 °C if not used immediately. Primers for *Hat1* (source: NCBI AK041700) and reference gene *Gapdh* (source: AC166162; Luo et al., 2024) were synthesized by Shanghai Biotech (sequences: *Hat1*-F: TGGAGTTAGTGAGAGGTTGGAAG, *Hat1*-R: TAGAGGTCAAGTCAGCAGTCAAG; *Gapdh*-F: GGTTGTCTCCTGCGACTTCA, *Gapdh*-R: TGGTCCAGGGTTTCTTACTCC). RT-qPCR was performed on a Light Cycler^®^ 96 System (Roche, Basel, Switzerland) using Hiff™ qPCR SYBR^®^ Green Master Mix (Yisheng Biotech, Shanghai, China). Reaction system (20 μl): 10 µl Master Mix, 0.4 µl each of 10 μM forward/reverse primer, 2 µl cDNA, RNase-Free ddH2O to volume. Cycling parameters: 95 °C for 30 s (initial denaturation), followed by 40 cycles of 95 °C for 10 s (denaturation) and 60 °C for 10 s (annealing/extension). Relative Hat1 mRNA expression was calculated using the 2ˆ(-ΔΔCt) method.

### Western blot analysis of Hat1 protein expression in mouse testes

Testis tissue (200 mg, 3/8-week-old mice) was lysed in RIPA buffer (Biyuntian Biotech, Shanghai, China) containing PMSF (Solebotide, Beijing, China), centrifuged at 12,000 rpm for 5 min at 4 °C, and supernatant was collected. Protein concentration was measured with a BCA Protein Assay Kit (Yazyme Biotech, Shanghai, China). Samples were mixed with 5 × protein loading buffer (Yase, Shanghai, China), denatured at 100 °C for 10 min, cooled on ice for 5 min, and centrifuged at 2,000 rpm for 1 min at 4 °C. SDS-PAGE electrophoresis (Yase, Shanghai, China) was performed at 80 V for 20 min, then 120 V for 60 min. Proteins were transferred to PVDF membranes (Millipore) *via* wet transfer (350 mA, 1.5 h, 4 °C), blocked with 5% skimmed milk (Biyuntian Biotech, Shanghai, China) for 1 h at room temperature (RT), and incubated with primary antibodies against Hat1/Gapdh (Able Antibody, Shanghai) (1:3000) followed by secondary antibodies (1:5000; Able Antibody, Shanghai, China). Signals were detected with the BeyoECL Kit (Beyotime, Shanghai, China) using a chemiluminescence imager; band intensities were quantified *via* ImageJ.

### Immunofluorescence analysis of hat1 protein in mouse testes

Fresh testis tissues were rinsed with saline, fixed in 4% paraformaldehyde for 12–24 h, dehydrated *via* graded alcohol (30%–100%, 1 h each; 70% overnight), cleared in xylene:anhydrous ethanol (1:1, 2 h) then xylene (twice, 1.5 h each), infiltrated with paraffin:xylene (1:1, 40 °C, overnight), and embedded (cold table solidification, 15–20 min). Sections (20–30 µm trimmed to 3–4 μm) were mounted on adhesive slides, warmed at 45 °C, and baked at 60 °C for 1–2 h. Dewaxing (xylene, twice 15 min) and rehydration (100% ethanol twice, then 90%/85%/75% ethanol, 5 min each; distilled water, 5 min) were performed. Antigen retrieval was done with retrieval solution in a microwave (medium-high heat 8 min, rest 8 min, medium-low heat 7 min). Slides were cooled, rinsed with PBS (3 ×5 min), circled with a histochemical pen, and blocked with 10% BSA (50 μl/slide, 1 h at RT). Sections were incubated overnight at 4 °C (light-protected wet chamber) with Hat1 (Cat. No. GB11906; Servicebio, Wuhan, China)/DDX4 antibodies (Cat. No. ab13840; Abcam, Shanghai, China; 1:200 in blocking solution/PBS), then with fluorescent secondary antibodies (1:200, 1 h at RT, dark; Able Antibody, Shanghai, China). DAPI (1:500 in PBS) was used for nuclear staining (15 min at RT). All steps included PBS washes (3 ×5 min). Sections were examined under a fluorescence microscope, and images were captured in the dark. The distribution and localization of Hat1 protein were observed, and fluorescence intensity was analyzed in different male germ cells using ImageJ software. To address potential artificial fluorescence intensity increases caused by dramatic chromatin condensation and cellular compaction during spermiogenesis (a key concern for germ cell immunofluorescence quantification), we used the formula: **Mean gray value = Integrated Density/Area**—a validated approach in spermatogenesis research to correct for tissue compaction-related artifacts (Liu et al., 2025; Nagamori, Cruickshank & Sassone-Corsi, 2011). For identification of different male germ cell types, spermatogonia, primary/secondary spermatocytes, round/elongating spermatids, and sperm were distinguished by their distinct distribution (along seminiferous tubules: basal → lumen) and morphology (*e.g.*, spermatogonia: small, round nuclei; sperm: tapered head + tail), as described previously (Her et al., 2021).

### Bioinformatics analysis of chromatin organization genes based on single-cell sequencing data

Genes involved in chromatin organization for the species *Mus musculus* were retrieved from QuickGO (https://www.ebi.ac.uk/QuickGO). Single-cell sequencing data (GSE214315) were sourced from the GEO dataset (https://www.ncbi.nlm.nih.gov/geo) and subjected to standardized preprocessing to ensure data comparability. To eliminate biases from sequencing depth and gene length, expression levels of all genes were normalized using the Reads Per Kilobase of transcript per Million mapped reads (RPKM) method. Subsequently, normalized data were used to identify differentially expressed genes (DEGs) during spermatogenesis. Significance was set at an FDR (false discovery rate) value <0.05 and —log_2_(FC)— ≥ 1 (FC represents fold change of gene expression in one sample compared to another). The individual cell types analyzed in the study included leptotene-zygotene cells, pachytene-diplotene cells, round spermatids, early elongating spermatids, and late elongating spermatids. The methods for identifying individual cell types refer to the reference of Gill et al. (2023); the original publication’s validation process meets the standards required for our research, and no additional proof of data reliability is necessary from our end.

### GO enrichment analysis of degs in chromatin organization

DEGs between adjacent phases of spermatogenesis were used for GO enrichment analysis with the DAVID v2023q4 tool (https://davidbioinformatics.nih.gov/). This aimed to theorize the molecular mechanisms of *Hat1* in conjunction with DEGs involved in chromatin organization and its potential regulatory functions in mouse spermatogenesis. GO term importance was determined using an FDR threshold <0.05, requiring at least 10 enriched genes for each set of GO terms, in both up- and down-regulated categories, when comparing different male germ cells.

### PPI analysis

PPI for Hat1 with the proteins encoded by DEGs in the BP of chromatin organization in the diversity male germ cells were analyzed using STRING (https://cn.string-db.org), with its default parameters and GO term significance set at an FDR value <0.05. This analysis aimed to construct PPI networks, elucidate Hat1’s specific functions and molecular mechanisms in chromatin organization, and hypothesize its regulatory roles in mouse spermatogenesis. By employing the STRING platform with its default parameters, our study leverages a robust and balanced approach to identify and analyze PPIs, ensuring that the results are both comprehensive and scientifically sound.

### Statistical analysis

Data analysis was performed using Statistical Package for the Social Sciences, version 23.0 (SPSS 23.0). Normally distributed data with equal variances were presented as mean ± standard deviation (*SD*). Two-sample comparisons were conducted using the two-tailed independent *t*-test, and multiple sample comparisons were made using one-way ANOVA. A *p*-value of <0.05 was considered statistically significant.

## Results

### Expression of Hat1 mRNA in the testes of 3-week-old and 8-week-old mice

**Figure 1:**
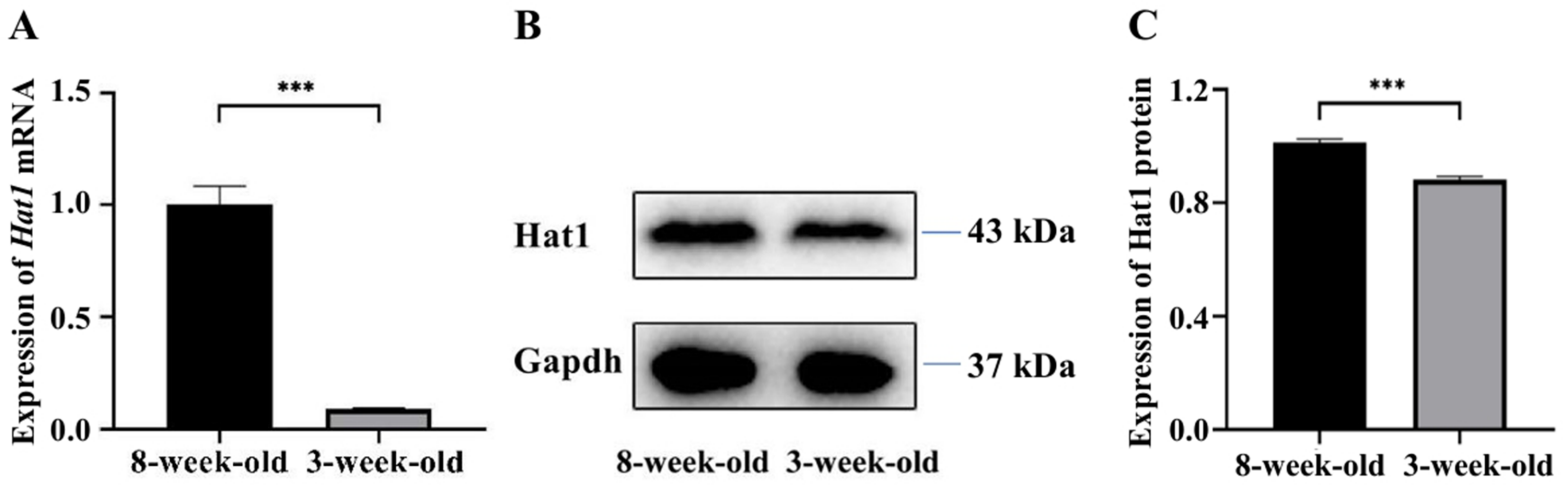
The expression of *Hat1* mRNA and protein in the testis tissues of 3-week-old and 8-week-old mice. (A) Expression of *Hat1* mRNA using RT-qPCR; (B) expression of Hat1 protein using WB; (C) relative expression of Hat1 protein based on result of WB. ^∗∗∗^*P* < 0.001.

RT-qPCR analysis revealed significant differential *Hat1* mRNA expression between 3-week-old and 8-week-old mouse testes (Fig. 1A). The relative expression level of *Hat1* mRNA in 8-week-old mice was 1.00 ± 0.09, while that in 3-week-old mice was 0.11 ± 0.01 (*P* < 0.05); the relative expression level of Hat1 mRNA in 8-week-old mice was approximately 9.0-fold higher than that in 3-week-old mice. Notably, 3-week-old mouse testes primarily contain immature germ cells (*e.g.*, spermatogonia, early primary spermatocytes) and underdeveloped somatic cells, while 8-week-old testes harbor fully mature germ cell populations (from spermatogonia to mature sperm). This age-dependent difference in germ cell maturity makes whole-tissue analysis informative for capturing developmental trends in Hat1 expression, as analogous studies have validated the use of whole testis samples to investigate age-related gene expression during spermatogenesis (Luo et al., 2024; Sun et al., 2022).

### Expression of hat1 protein in the testes of 3-week-old and 8-week-old mice

WB analysis confirmed the mRNA expression trend (Figs. 1B, 1C), and quantitative analysis of WB bands showed that the relative expression level of Hat1 protein in 8-week-old mouse testes was 1.03 ± 0.02, while that in 3-week-old mice was 0.86 ± 0.02 (*P* < 0.05). The Hat1 protein level in 8-week-old mice was approximately 1.3-fold higher than that in 3-week-old mice, consistent with the transcriptional level changes and suggesting that Hat1 expression is regulated at both mRNA and protein levels during mouse sexual maturation.

### Spatial distribution and stage-specific expression of Hat1 protein in mouse germ cells

**Figure 2:**
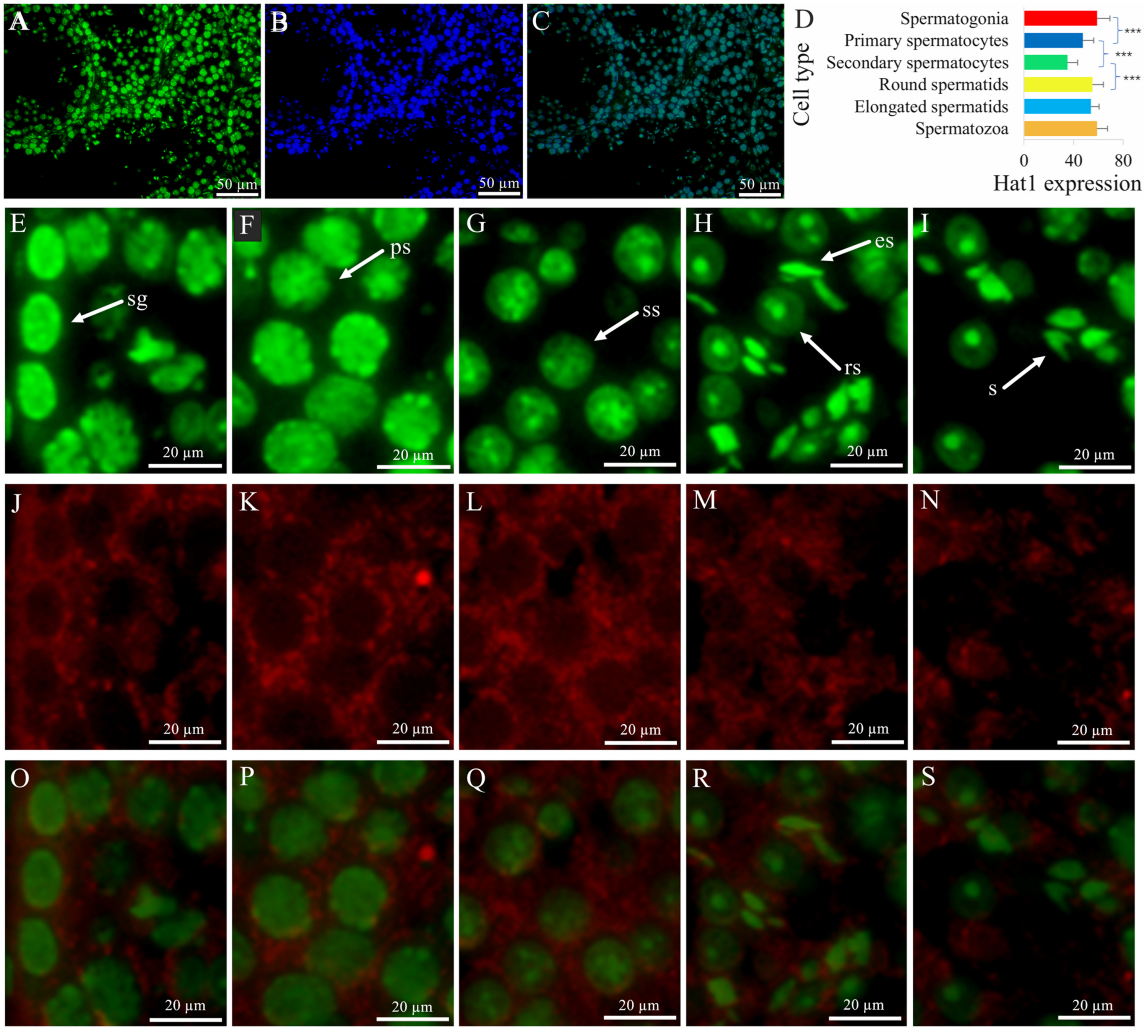
The expression and location of Hat1 protein in the mouse testes. DDX4 is a marker protein of male germ cells, mainly distributed in the cytoplasm. (A) Hat1; (B) DNA; (C) merging of (A) and (B); (D), comparison of Hat1 expression in the different male germ cells in the mouse testes; (E) Hat1 in spermatogonia; (F) Hat1 in primary spermatocytes; (G) Hat1 in secondary spermatocytes; (H) Hat1 in both round elongating spermatids; (I) sperm; (J) Ddx4 in spermatogonia; (K) Ddx4 in primary spermatocytes; (L) Ddx4 in secondary spermatocytes; (M) Ddx4 in both round elongating spermatids; (N) Ddx4 sperm; (O) merging of (E) and (J); (P) merging of (F) and (K); (Q) merging of (G) and (L); (R) merging of (H) and (M); (S) merging of (I) and (N). sg, spermatogonia; ps, primary spermatocytes, ss, secondary spermatocytes; rs, round spermatids; es, elongating spermatids; s, sperm. each scale bar in the images of A, B, and C is 50 µm; each scale bar in the images of E–S is 20 µm. *** *P* < 0.001 compared between the different male germ cells during the adjacent phases of spermatogenesis.

IF localization showed that Hat1 protein was predominantly localized in the nuclei of all male germ cell types, with distinct expression patterns across developmental stages (Fig. 2). DDX4, a specific marker for male germ cells, was used to verify the cell type specificity of Hat1 expression: The merged co-localization image of Hat1 (green fluorescence, nuclear localization) and DDX4 (red fluorescence, cytoplasmic localization), clearly showing overlapping signals in germ cells—confirming that Hat1 is specifically expressed in male germ cells.

Semi-quantitative analysis (using the corrected Mean gray value = Integrated Density/Area, see Methods) revealed that the Hat1 expression levels appeared variable at the male germ cell development stages, with the high level in the spermatogonia (58.86 ± 10.46, *n* = 15), round spermatids (55.30 ± 8.70, *n* = 22), and sperm (58.82 ± 8.55, *n* = 25), the intermediate in the primary spermatocytes (47.37 ± 8.86, *n* = 29), and the low in secondary spermatocytes (35.12 ± 8.18, *n* = 30) (Fig. 2D). The calculations based on these values revealed key fold relationships: Primary spermatocytes showed Hat1 fluorescence intensity ∼0.8-fold that of spermatogonia; secondary spermatocytes had ∼0.7-fold the fluorescence intensity of primary spermatocytes; round spermatids exhibited ∼1.6-fold higher fluorescence intensity than secondary spermatocytes. Notably, weak non-specific DDX4 signals were observed in Leydig cells, which are attributed to technical artifacts caused by antibody cross-reactivity (a common phenomenon in immunofluorescence assays for germ cell markers). This non-specific signal does not interfere with the identification of Hat1-expressing germ cells, as germ cells can be further distinguished by their typical morphology (*e.g.*, spermatogonia: basal localization in seminiferous tubules, small round nuclei) and co-localization with Hat1. The IF-derived quantitative expression trend is consistent with WB results (higher Hat1 protein in 8-week-old *vs.* 3-week-old testes), reinforcing the reliability of the current data. While WB analysis of isolated germ cell populations would further validate cell-type-specific Hat1 levels, resource constraints necessitate this as a priority for future work (Her et al., 2021). Combined with cell morphology, DDX4 co-localization, and nuclear localization (a hallmark of Hat1’s chromatin-related function), the current IF data still reliably reflects the stage-specific expression of Hat1 during spermatogenesis. During the transition between adjacent stages, Hat1 expression was significantly downregulated from spermatogonia to primary spermatocytes, and from primary to secondary spermatocytes (*P* < 0.05); It was significantly upregulated from secondary spermatocytes to round spermatids (*P* < 0.05); No significant difference was observed between round spermatids and sperm, indicating that Hat1 maintains high expression during late spermiogenesis and in mature sperm.

### Identification of chromatin organization-related differentially expressed genes (DEGs)

**Figure 3:**
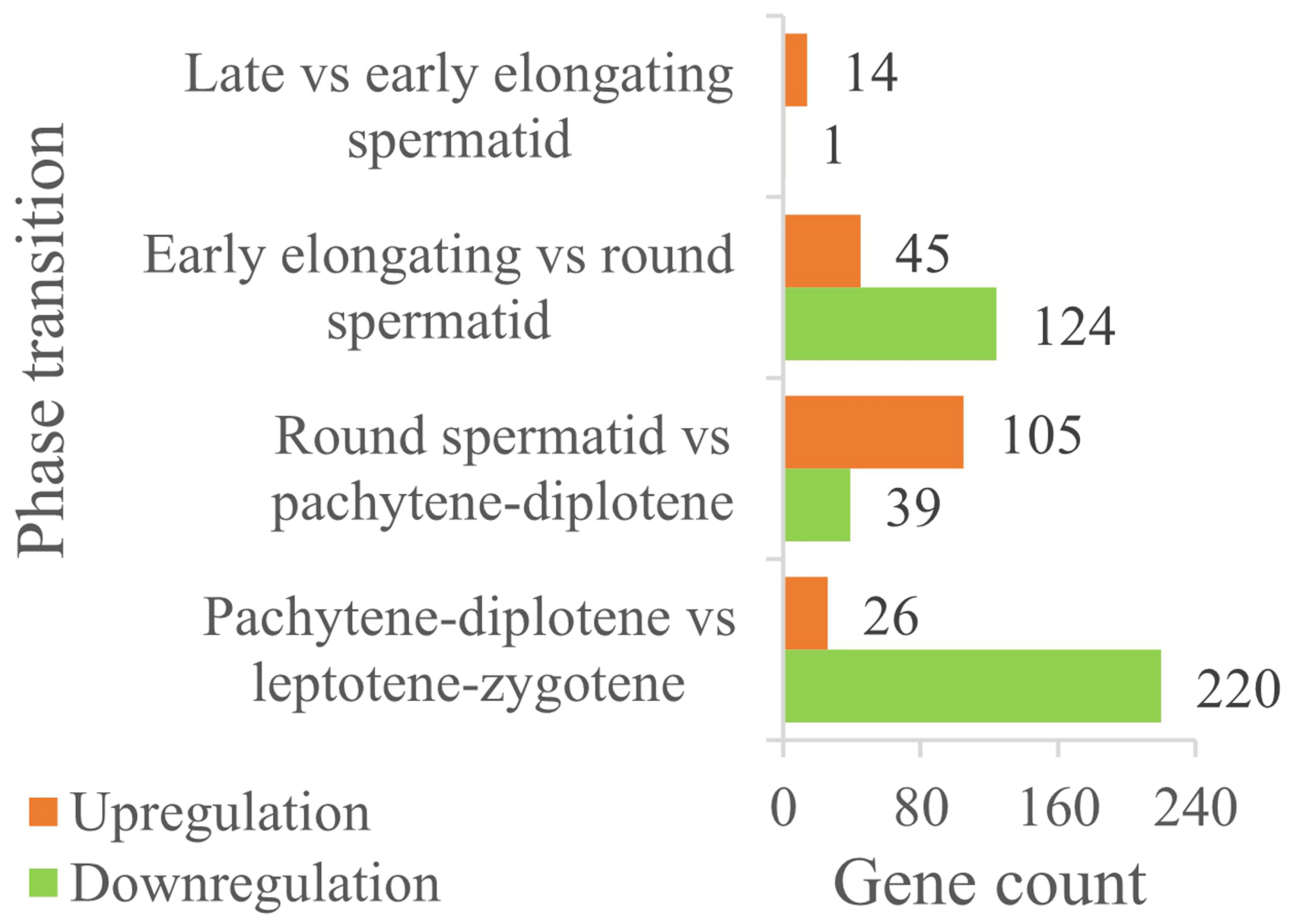
The expression comparison of DEGs in chromatin organization between the different male germ cells during the adjacent phases of spermatogenesis in the mouse testes.

A total of 835 chromatin organization-related proteins were retrieved from QuickGO. Single-cell sequencing data (GSE214315) analysis identified 246 protein-coding DEGs (FDR < 0.05, | flog_2_(FC)| ≥ 1) across key germ cell stages (Fig. 3). Stage-specific DEG distribution included:

(1) pachytene-diplotene *vs.* leptotene-zygotene: 220 downregulated DEGs;

(2) round spermatids *vs.* early elongating spermatids: 124 downregulated DEGs;

(3) round spermatids *vs.* pachytene-diplotene: 105 upregulated DEGs;

(4) notably, no significant DEGs were detected between late and early elongating spermatids.

Importantly, these bioinformatics findings are mutually verified with experimental results: for example, single-cell data shows high Hat1 expression in mature germ cells (*e.g.*, round spermatids, sperm)—a trend consistent with RT-qPCR/WB results (higher Hat1 in 8-week-old testes, which are enriched in mature germ cells). This cross-validation strengthens the robustness of the current DEG analysis.

### Functional enrichment of chromatin organization-related DEGs

**Figure 4:**
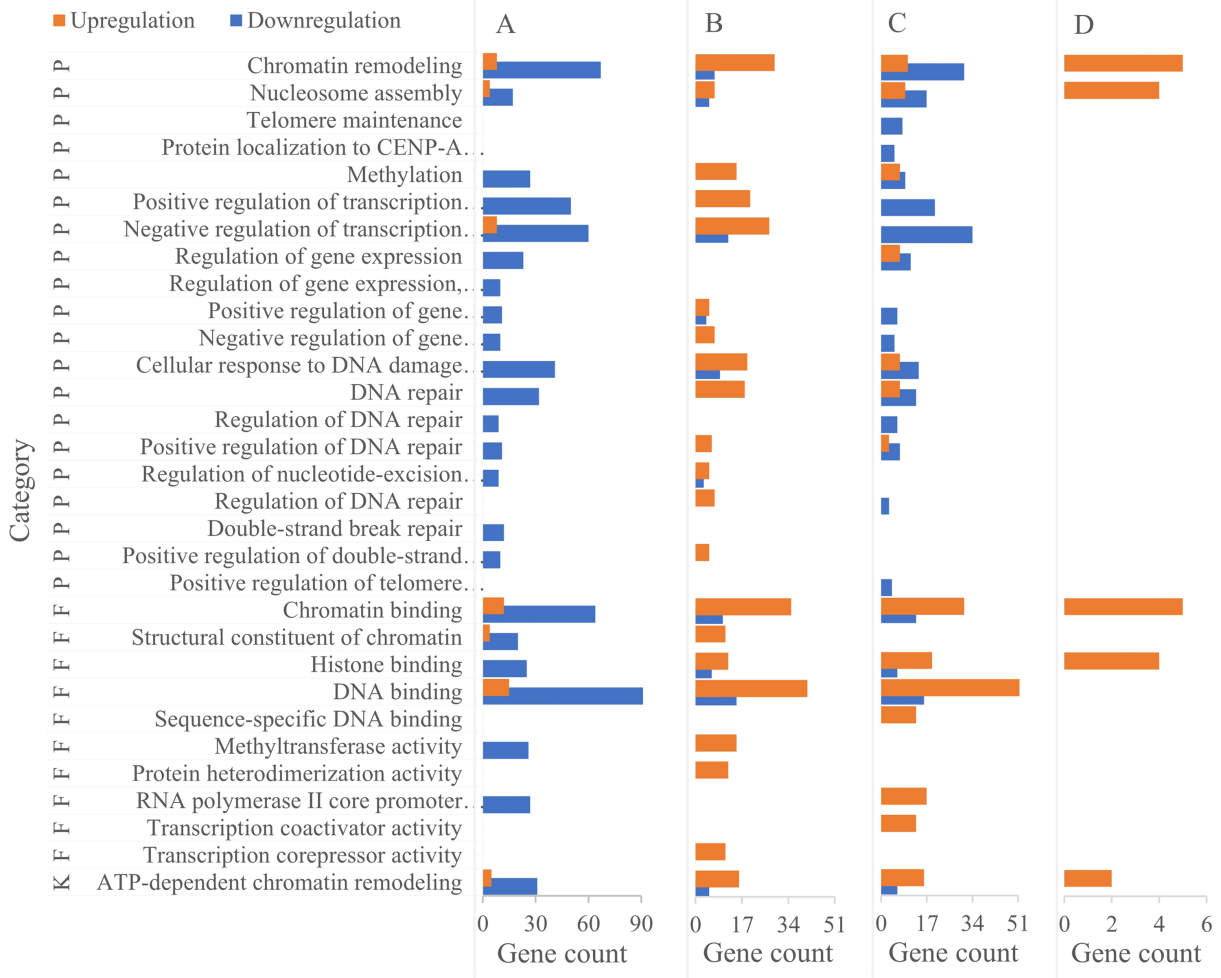
Functional enrichment of DEGs in chromatin organization between different male germ cells in the mouse testes. (A) Pachytene-diplotene *vs* leptotene-zygotene; (B) round spermatid *vs* pachytene-diplotene; (C) early elongating *vs* round spermatid; (D) late *vs* early elongating spermatid. P, biological process; K, KEGG pathway; F, molecular function.

DAVID-based GO enrichment analysis of chromatin organization-related DEGs revealed distinct functional patterns that align with the biological roles of specific germ cell types. To enhance clarity, Fig. 4 has been reorganized by cell type (primary/secondary spermatocytes, round spermatids, elongating spermatids), directly linking DEG-enriched functions to the unique demands of each cell type during spermatogenesis. The key findings are as follows:

(1) primary/secondary spermatocytes: DEGs were predominantly enriched in biological processes (BPs) related to transcription regulation, including positive/negative regulation of RNA polymerase II-mediated transcription. This enrichment supports the active transcriptional program required for meiotic initiation and homologous chromosome pairing—core functions of spermatocytes during meiosis.

(2) round spermatids: Enrichment shifted toward refined epigenetic transcriptional regulation and chromatin assembly. These functions are critical for the extensive cellular metamorphosis of round spermatids (*e.g.*, nuclear condensation, cytoplasmic restructuring) as they transition to elongating spermatids, laying the foundation for subsequent sperm maturation.

(3) elongating spermatids: DEGs were enriched in two key BPs: DNA repair and ATP-dependent chromatin remodeling. The DNA repair enrichment contributes to protecting sperm genetic material from damage during late spermiogenesis, while the ATP-dependent chromatin remodeling pathway supports the final stages of nuclear compaction—both essential for forming functionally competent sperm.

Notably, the ATP-dependent chromatin remodeling pathway was enriched in all three cell types except between late and early elongating spermatids, with variations in DEG types and quantities reflecting dynamic adjustments to chromatin dynamics across developmental transitions.

### Hat1-interacting proteins and their functional characteristics

**Figure 5:**
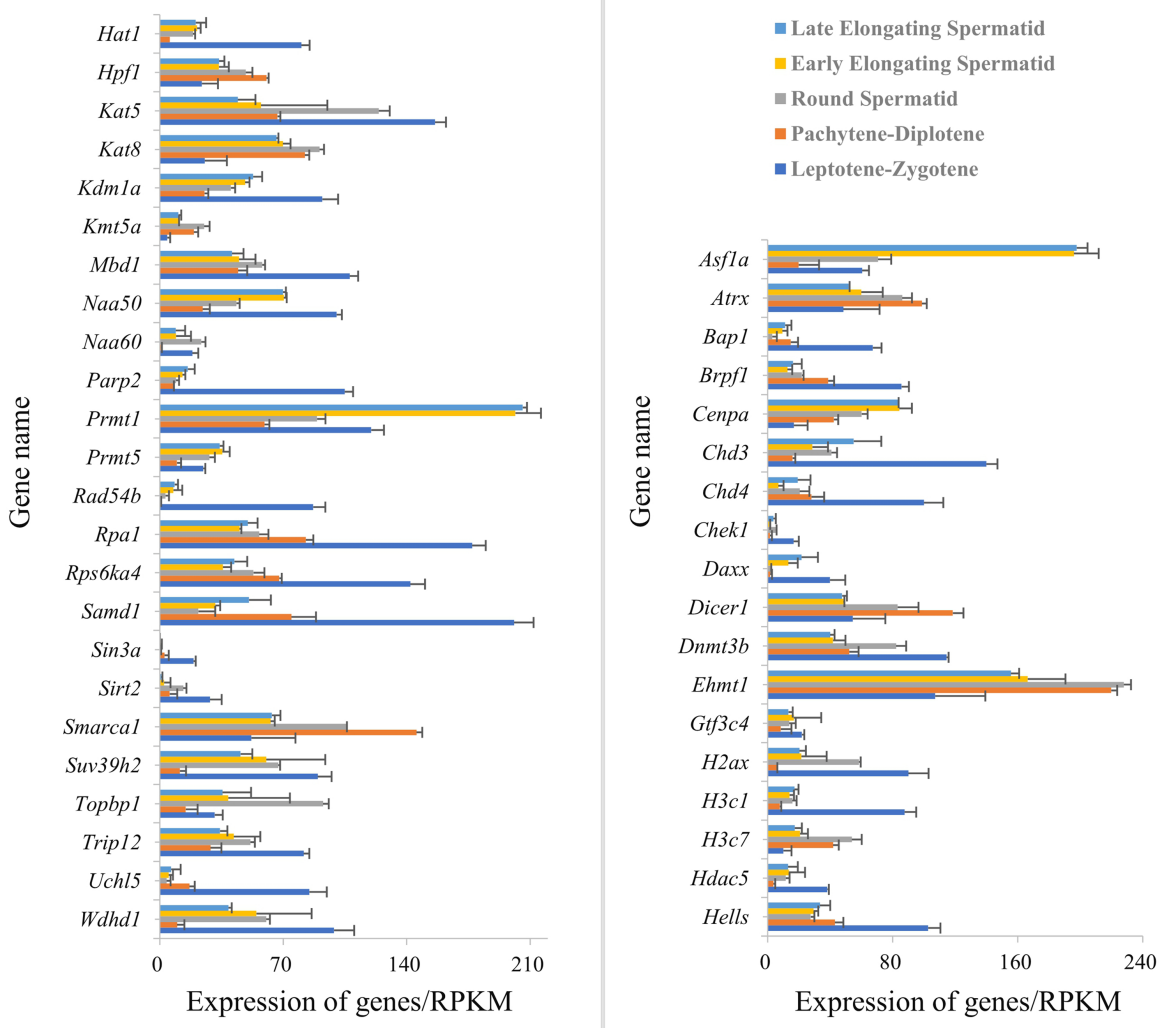
Expression of *Hat1* and DEGs encoding Hat1-interacting proteins in chromatin organization in the mouse testes.

PPI analysis (STRING) constructed a network of 246 chromatin organization-related DEG-encoded proteins, among which 41 were identified as Hat1-interacting proteins (Fig. 5). All expression data of these 41 interactors were derived from the RPKM-normalized single-cell sequencing data (GSE214315)—ensuring consistent quantification standards across different germ cell stages and avoiding biases from sequencing depth or gene length. These interactors showed stage-specific mRNA expression patterns (upregulated or downregulated) across spermatogenesis.

**Figure 6:**
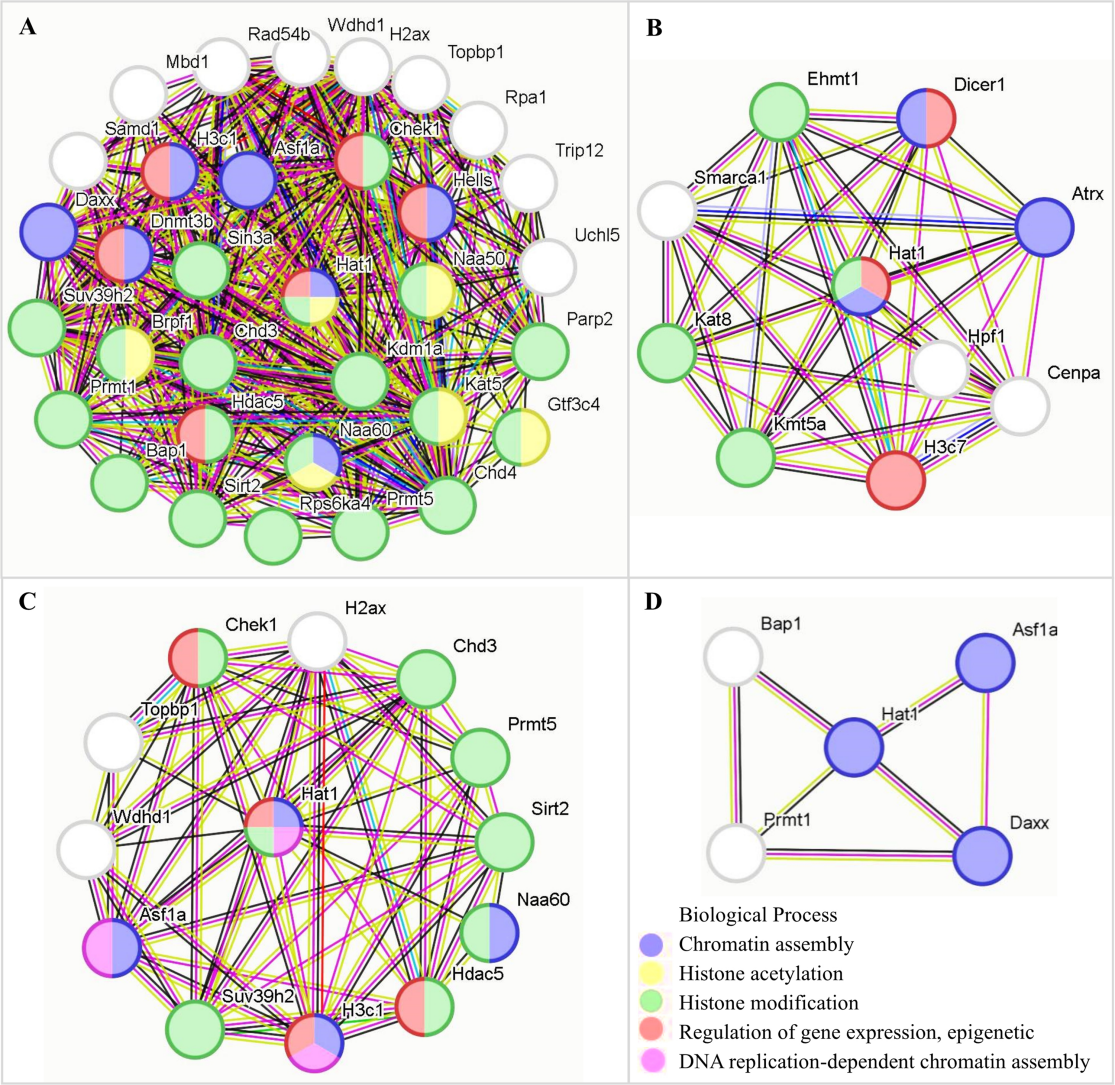
The functions of Hat1 with Hat1-interacting proteins encoded by DEGs in chromatin organization in the mouse testes. (A) Downregulation during the pachytene-diplotene phase *vs* the leptotene-zygotene phase (or upregulation during the leptotene-zygotene phase *vs* the pachytene-diplotene phase); (B) upregulation during the pachytene-diplotene phase *vs* the leptotene-zygotene phase; (C) upregulation in the round spermatid phase *vs* the pachytene-diplotene phase; (D) upregulation in the early elongating spermatid phase *vs* the round spermatid phase.

Stage-specific GO enrichment of Hat1-interacting proteins (Fig. 6) showed that:

(1) leptotene-zygotene to pachytene-diplotene: The histone acetylation BP (enriched for Hat1 and its interactors) was significantly downregulated;

(2) round spermatid phase: Hat1 and interactors (*e.g.*, Wdhd1, Topbp1) were enriched in BPs including chromatin assembly, histone acetylation, epigenetic regulation, and DNA replication-dependent chromatin assembly (no downregulated interactors detected);

(3) round to early elongating spermatids: Upregulated interactors (*e.g.*, Prmt1, Daxx) converged on the “chromatin assembly” GO term (no enriched terms for downregulated interactors).

The current PPI data, paired with experimental observations of Hat1’s localization/expression, provides a preliminary but valuable framework for understanding Hat1’s regulatory network in chromatin organization.

## Discussion

The present study comprehensively explored the expression dynamics and functional significance of Hat1 in mouse spermatogenesis, with a particular focus on its role in orchestrating chromatin organization. By integrating results from RT-qPCR, WB, IF localization, and multi-dimensional bioinformatics analyses (including DEG screening, GO enrichment, and PPI network construction), we uncovered stage-specific regulatory patterns of Hat1 and its interplay with chromatin-related molecular networks. These findings not only fill gaps in our understanding of Hat1’s function in male germ cell development but also align with and extend previous observations on epigenetic regulation during spermatogenesis.

### Age-dependent and stage-specific expression of Hat1: functional adaptation to spermatogenic demands

A pivotal finding was the significant upregulation of both Hat1 mRNA and protein in the testes of 8-week-old sexually mature mice compared to 3-week-old pre-pubertal mice. This age-dependent expression pattern is consistent with prior reports of stage-specific gene modulation during testicular development (Wu et al., 2022; Luo et al., 2024), as 8-week-old mice exhibit fully established spermatogenesis—characterized by the continuous progression of germ cells from spermatogonial stem cells to mature sperm—whereas 3-week-old mice undergo early testicular differentiation with limited germ cell maturation (*e.g.*, fewer round spermatids and no mature sperm). This temporal correlation strongly suggests that Hat1 is functionally coupled to the execution of mature spermatogenic processes, particularly those requiring precise chromatin remodeling to support cell proliferation, meiosis, and sperm maturation.

Complementary IF analysis further revealed that Hat1 is predominantly localized to the nuclei of all male germ cell types, a distribution that aligns with its identity as a histone acetyltransferase (HAT) involved in chromatin structure modulation and gene expression regulation (Liu et al., 2025; Oishi et al., 2023). The stage-specific expression profile of Hat1—peaking in spermatogonia and sperm, exhibiting intermediate levels in primary spermatocytes, and reaching the lowest in secondary spermatocytes—reflects its adaptive roles across distinct germ cell developmental stages. Notably, the identification of spermatogonia in our IF analysis relied on well-established morphological criteria which is not arbitrary but aligns with the characteristics of mouse spermatogonia reported by Gill et al. (2023) in their comprehensive analysis of stage-specific transcriptomes in male germ cells. Gill et al. (2023) explicitly linked these morphological features to spermatogonial identity, validating their use as reliable markers for distinguishing spermatogonia from other testicular cell types (*e.g.*, Sertoli cells, which localize to the seminiferous tubule epithelium but lack the “small round nuclei” of spermatogonia). Given the consistency between our morphological identification and the literature (Gill et al., 2023), combined with Hat1’s nuclear localization (a hallmark of its chromatin-related function), our conclusion regarding high Hat1 expression in spermatogonia remains robust.

In spermatogonia, high Hat1 expression likely sustains the active transcriptional program necessary for mitotic proliferation and maintenance of stem cell properties (Liu et al., 2010). As noted by An et al. (2015), histone modifications (including acetylation) play critical roles in maintaining chromatin accessibility for gene expression during spermatogonial division, and Hat1’s enrichment here may facilitate the biosynthesis of proteins required for cell cycle progression. In contrast, the marked downregulation of Hat1 in secondary spermatocytes correlates with the transient meiotic phase, where chromatin condensation takes precedence over transcriptional activity to ensure accurate chromosome segregation (Ishiguro & Shimada, 2022; Eyster et al., 2019). During this stage, the priority shifts from gene expression to structural chromatin changes, reducing the need for Hat1-mediated acetylation.

Last but not least, Hat1 maintained high expression levels in round spermatids, elongating spermatids, and mature sperm. In mature sperm, where transcriptional activity is drastically diminished due to extensive chromatin condensation (Meyer-Ficca et al., 2013), this persistent high expression is unlikely to mediate transcription. Instead, it may contribute to two key processes: (1) protecting sperm genetic material, as supported by our bioinformatics findings linking Hat1 to the BP of DNA repair (Minami, Iida & Maeshima, 2022), and (2) maintaining chromatin accessibility at specific loci *via* histone acetylation (Wu et al., 2022; Jha et al., 2017)—a function critical for post-fertilization chromatin decondensation and embryonic genome activation. While these hypotheses require further validation, they highlight Hat1’s multifaceted roles beyond canonical transcriptional regulation.

### Chromatin organization-related DEGs and Hat1-interacting proteins: a dynamic regulatory network

Bioinformatics analysis of single-cell sequencing data (GSE214315) identified 246 DEGs associated with chromatin organization across key spermatogenic stages, alongside 41 Hat1-interacting proteins encoded by these DEGs. This network underscores the dynamic modulation of chromatin dynamics to meet the unique functional demands of each germ cell stage, as previously emphasized by Gill et al. (2023) in their analysis of stage-specific transcriptomes in mouse male germ cells.

During the leptotene-zygotene phase—when homologous chromosomes pair and synaptonemal complexes form (Dunleavy & Collins, 2017; Shibuya, Morimoto & Watanabe, 2014)—Hat1 and its interacting proteins were enriched in BPs related to transcription regulation (*e.g.*, positive/negative regulation of RNA polymerase II-mediated transcription). This enrichment is critical for initiating meiosis, as it facilitates the biosynthesis of proteins required for homologous recombination and chromosome synapsis. As spermatogenesis progresses to the round spermatid phase, a notable shift occurred toward GO terms associated with refined epigenetic transcriptional regulation and chromatin assembly. This transition aligns with the extensive cellular metamorphosis of round spermatids into elongating spermatids, a process requiring precise chromatin remodeling to prepare for nuclear condensation (Liu et al., 2021; Mishra et al., 2018; Kutchy et al., 2017). For instance, Hat1-interacting proteins such as Wdhd1 and Topbp1 were enriched in BPs like chromatin assembly and DNA replication-dependent chromatin assembly, highlighting their role in organizing chromatin structure for subsequent differentiation.

The ATP-dependent chromatin remodeling pathway—enriched in all adjacent spermatogenic stages except between late and early elongating spermatids—further emphasizes the dynamic nature of chromatin regulation. Stage-specific differences in DEG distribution within this pathway (*e.g.*, upregulation of NURD, NURF, and BAF complexes in round spermatids) suggest that distinct chromatin remodeling complexes are recruited to mediate stage-specific functions, such as chromatin condensation or gene silencing (Wang et al., 2019). This aligns with Ortega et al. (2023) and Xu et al. (2023), who reported that Hat1 modulates chromatin conformation through interactions with remodeling complexes, underscoring a conserved mechanism across cell types.

Notably, our findings are consistent with cross-species studies: Liu et al. (2025) observed higher Hat1 expression in mature testes of Chinese mitten crabs (*Eriocheir sinensis*) compared to juveniles, while Sun et al. (2022) reported differential Hat1 expression in yak testes—suggesting potential conserved roles of Hat1 in spermatogenesis across taxa. This conservation highlights the fundamental importance of Hat1-mediated chromatin organization in male germ cell development.

### Mechanistic insights, implications for reproductive biology and study limitations

The stage-specific enrichment of Hat1 and its interactors in diverse BPs—from transcription regulation (leptotene-zygotene) to epigenetic modulation (round spermatids) and DNA repair (late spermiogenesis)—highlights Hat1’s role as a central coordinator of chromatin organization during spermatogenesis. For example, during the transition from round to early elongating spermatids, upregulated Hat1-interacting proteins (*e.g.*, Prmt1, Daxx) converged on the “chromatin assembly” GO term, suggesting a focused role in stabilizing chromatin structure as spermatids elongate. In contrast, the downregulation of histone acetylation-related BPs during the pachytene-diplotene phase may reflect a shift toward chromatin condensation to support meiotic division (Ishiguro & Shimada, 2022), with Hat1’s reduced expression aligning with this structural priority.

This study advances our understanding of the epigenetic regulation of spermatogenesis by delineating Hat1’s stage-specific functions in chromatin organization. The precise control of Hat1 expression and its integration into chromatin-related networks underscores the importance of coordinated epigenetic modulation for male germ cell development—from mitotic proliferation of spermatogonia to the formation of genetically intact sperm. These findings have broader implications for reproductive biology: aberrant Hat1 expression or function could disrupt chromatin organization, leading to impaired spermatogenesis, reduced sperm quality, or male infertility. For example, if Hat1’s role in DNA repair (Minami, Iida & Maeshima, 2022) is compromised, it may increase the risk of genetic mutations in sperm, contributing to embryonic developmental defects.

Though the correlations identified in this study hold significant reference value, there remains room for further exploration in terms of research depth and mechanistic investigation. Subsequent studies could conduct functional experiments (*e.g.*, Hat1 knockout or knockdown in mouse germ cells), combining *in vitro* germ cell culture and *in vivo* genetic models to clarify the causal relationship between Hat1 expression and spermatogenic processes—such as verifying whether reduced Hat1 expression impacts meiotic progression, chromatin condensation, or sperm DNA integrity. Additionally, techniques like co-immunoprecipitation (Co-IP) or proximity ligation assays may help dissect the molecular mechanisms underlying Hat1’s interactions with its 41 partner proteins, including whether these interactions are direct or mediated by co-factors and how they regulate chromatin dynamics.

In summary, this study establishes Hat1 as a critical regulator of mouse spermatogenesis, with stage-specific functions mediated through chromatin organization. By unraveling the spatiotemporal and molecular dynamics of Hat1, our findings lay the groundwork for future investigations into the epigenetic basis of male fertility and potential therapeutic targets for male infertility.

## Conclusion

**Figure 7:**
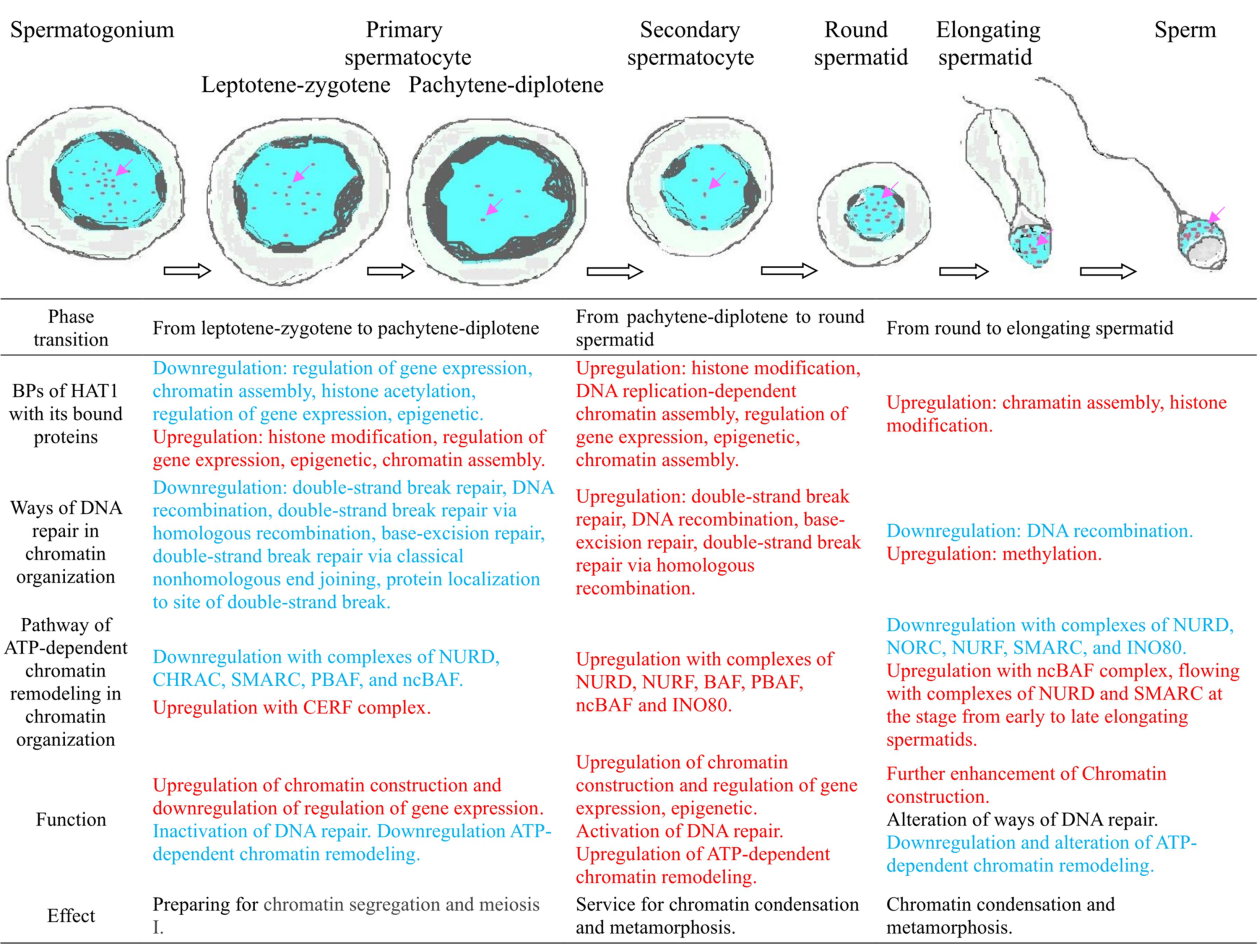
The expression of Hat1 and the mechanisms of Hat1 with DEGs in chromatin organization involved in the mouse spermatogenesis based on both the results of immunofluorescence localization and the analysis of single-cell sequencing data (GSE214315). Blue represents nuclei and purple dots represent Hat1.

The research presented a detailed analysis of Hat1 expression and its functional implications across the stages of spermatogenesis in the mice, utilizing methods, such as RT-qPCR, WB, immunofluorescence localization, and bioinformatics. We found that both Hat1 mRNA and protein were highly expressed in the testes of 8-week-old mice compared to those of 3-week-old mice. Hat1 protein was prominently expressed in the nuclei of various male germ cell types, with significant expression differences observed between specific cell types. The expression of Hat1 was highest in spermatogonia and sperm, followed by primary spermatocytes, and was lowest in secondary spermatocytes. Throughout the spectrum of spermatids and sperm, Hat1 maintained high expression levels. Bioinformatics analysis and functional enrichment showed that a large number of DEGs in chromatin organization and a total of 41 Hat1-interacting proteins encoded by the DEGs were expressed in male germ cells at different stages, and they were enriched in different GO terms at various stages. During the leptotene-zygotene phase, *Hat1* and other chromatin organization genes were predominantly involved in the regulation of transcription, facilitating the initial steps of meiosis. As spermatogenesis progresses to the round spermatid phase, there was a marked shift towards a refined epigenetic transcriptional regulation and chromatin assembly, essential for the subsequent stages of cellular metamorphosis and sperm maturation. Additionally, both the BP of DNA repair and the pathway of ATP-dependent chromatin remodeling in chromatin organization at the late stages of spermiogenesis and within the sperm also participated in the protection of the sperm genetic material and transcript regulation through distinct mechanisms. It was seen that Hat1 had prominent functions in mouse sperm, but the functions it participated in through chromatin organization varied at different stages of male germ cell development based on the results of GO enrichment. In summary, the research provided valuable insights into the complex regulatory mechanisms governing chromatin organization during spermatogenesis (Fig. 7). The stage-specific expression patterns of Hat1 and its interactors highlighted the importance of precise control of gene expression and chromatin remodeling, as well as DNA repair in protection of sperm genetic material, in the development of male germ cells.

## Supplemental Information

10.7717/peerj.20240/supp-1 Supplemental Information 1 ARRIVE 2.0 Checklist

10.7717/peerj.20240/supp-2 Supplemental Information 2 ARRIVE 2.0 Checklist: Pre-Study Protocol Declaration

10.7717/peerj.20240/supp-3 Supplemental Information 3 RT-qPCR-tyl20210302-Hat1-mouse

10.7717/peerj.20240/supp-4 Supplemental Information 4 Immunofluorescence 1

10.7717/peerj.20240/supp-5 Supplemental Information 5 Immunofluorescence 2

10.7717/peerj.20240/supp-6 Supplemental Information 6 Immunofluorescence 3

10.7717/peerj.20240/supp-7 Supplemental Information 7 Immunofluorescence 4

10.7717/peerj.20240/supp-8 Supplemental Information 8 WB Expression of Hat1 protein 1

10.7717/peerj.20240/supp-9 Supplemental Information 9 WB Expression of Hat1 protein 2

10.7717/peerj.20240/supp-10 Supplemental Information 10 WB whole film

10.7717/peerj.20240/supp-11 Supplemental Information 11 Codebook

## Additional Information and Declarations

### DNA Deposition

The following information was supplied regarding the deposition of DNA sequences: The gene data is available at GenBank: 107435.

### Data Availability

The following information was supplied regarding data availability: The raw data is available in the Supplemental Files.
