# Supplementary material for: Deciphering the role of Hat1 in spermatogenesis: Chromatin organization and beyond
Source: PeerJ. 2025 Nov 19;13:e20240. doi: 10.7717/peerj.20240 (PMC12640128; doi:10.7717/peerj.20240)
Supplement: Supplemental Information 2 [file peerj-13-20240-s002.pdf]

Pre-Study Protocol Declaration

1. Study Title

Deciphering the role of Hat1 in spermatogenesis: Chromatin organization and beyond

2. Investigators

Principal Investigator Name: Genliang Li, [Youjiang Medical University for Nationalities]

Co-Investigator Names：Shenni Peng, Yulian Tang, Ruiqun Lu , Shi Huang , Yinyin Mo ,Hailing Huang [Youjiang Medical University for Nationalities]

3. Key Design Features

3.1 Type of research: experimental

3.2 Sample size justification: n≥3 is a common lower limit for biological replicates to ensure reproducible results. In this experiment, the number of mice in each experimental group was set at n=5 to minimize the experimental error, taking into account resource constraints (e.g., funding, personnel, experimental site, etc.).

3.3 Inclusion/exclusion criteria:

Inclusion criteria: The experimental groups were required to be normal mice 3 weeks old (12.5 ± 0.5 g) and 8 weeks old (27.5 ± 2.5 g) healthy male mice with no physical injuries or diseases and in good mental status.

Exclusion criteria： Mice were not within the range of 3-week-old (12.5 ± 0.5 g) and 8-week-old (27.5 ± 2.5 g) weights, had injuries, and were in poor mental condition.

3.4 Background and Rationale

Spermatogenesis, essential for male fertility, involves chromatin organization mediated by histone acetyltransferases (Hats)This research focused on exploring the spatiotemporal dynamics and regulatory network of Hat1 during the mouse spermatogenesis and analyzing molecular mechanism of Hat1 involved in the mouse spermatogenesis by BP of chromatin organization, using the methods of RT-qPCR, western bolt, immunofluorescence localization, bioinformatics, etc.

3.5 Objectives

Primary Objective:

This research focused on exploring the spatiotemporal dynamics and regulatory network of Hat1 during the mouse spermatogenesis and analyzing molecular mechanism of Hat1

spermatogenesis

Secondary Objectives:

To determine the expression pattern and localization of Hat1 during different stages of spermatogenesis

To investigate Hat1's impact on histone modification patterns in germ cells

To analyze Hat1's role in chromatin organization during spermatogenesis

3.6 Study Design

A laboratory study using normal mice as a model.

3.7. Materials and Methods

3.8 Animal Models

Healthy male mice, aged 3 weeks (12.5 ± 0.5 g) and 8 weeks (27.5 ± 2.5 g),

4. Sample Collection

Testes will be collected and processed for:

RNA extraction (RT-qPCR)

Protein extraction (Western blot)

Histological sections (Immunofluorescence)

5.Bioinformatics Analysis

Bioinformatics Analysis of Chromatin Organization Genes Based on Single-Cell Sequencing Data

DEGs between adjacent phases of spermatogenesis were used for GO enrichment analysis with the DAVID v2023q4 tool (https://david.ncifcrf.gov).

Prediction of protein-protein interaction networks using STRING database

6. Molecular Biology Techniques

RT-qPCR:

RNA extraction using TRIzol method

cDNA synthesis and quantitative PCR with SYBR Green

The relative mRNA expression levels of *Hat1* were calculated using the ΔΔCt method.

Western Blot:

Protein extraction using RIPA buffer

SDS-PAGE electrophoresis and transfer to PVDF membranes

Antibodies: anti-Hat1 (primary), HRP-conjugated secondary

Detection using chemiluminescence and quantification by densitometry

7. Immunofluorescence (IF)

Testis paraffin sections

Antigen retrieval (if needed)

Primary antibodies: Hat1, stage-specific markers

Secondary antibodies with fluorescent conjugates

Nuclear staining with DAPI

Treated Sections were examined under a fluorescence microscope, and images were captured in the dark.

8. Data Analysis Plan

Data analysis was performed using Statistical Package for the Social Sciences, version 23.0 (SPSS 23.0). Normally distributed data with equal variances were presented as mean ± standard deviation (SD). Two-sample comparisons were conducted using the two-tailed independent t-test, and multiple sample comparisons were made using one-way ANOVA. A p-value of <0.05 was considered statistically significant.

All experiments will include at least 3 biological replicates

9. Expected Outcomes

Hat1 played significant roles in mouse sperm, with its functions through chromatin organization varying across different stages of male germ cell development. Comprehensive expression profile of Hat1 during spermatogenesis. Identification of Hat1-dependent chromatin changes in germ cells. Characterization of Hat1's role in meiotic and post-meiotic stages.

10. Potential Limitations

Epigenetic variability between individual animals

11. Ethical Considerations

The study was approved by the Experimental Animal Ethics Committee of Youjiang Medical College for Nationalities with the approval number [2024032901]. All methods in the study were performed in accordance with the relevant guidelines and regulations of Youjiang Medical College for Nationalities (SYXK(Gui)2017-0004).

12. Protocol Registration

Not Registered: There was no registration for this trial, and due to time constraints, this study was not prospectively registered in the program registry.All methods and analyses were pre-planned and recorded before data collection to minimize bias. This experimental study involved animal experiments and has been approved by the Experimental Animal Ethics Committee of the Righteous Medical College of Nationalities under the approval number [2024032901]. All methods in the study were performed in accordance with the relevant guidelines and regulations of the Youjiang Medical College of Nationalities (SYXK(Gui)2017-0004).We declare that this study will be conducted according to the described protocol and all applicable regulations.

Any modifications will be documented and approved by the relevant oversight committees.
