# Supplementary figures and images for: Deciphering the role of Hat1 in spermatogenesis: Chromatin organization and beyond
Source: PeerJ. 2025 Nov 19;13:e20240. doi: 10.7717/peerj.20240 (PMC12640128; doi:10.7717/peerj.20240)

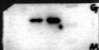

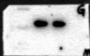

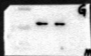

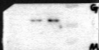

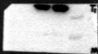

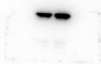

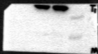

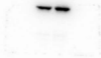

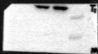

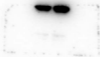

Supplement: Supplemental Information 8 [file peerj-13-20240-s008.pdf]

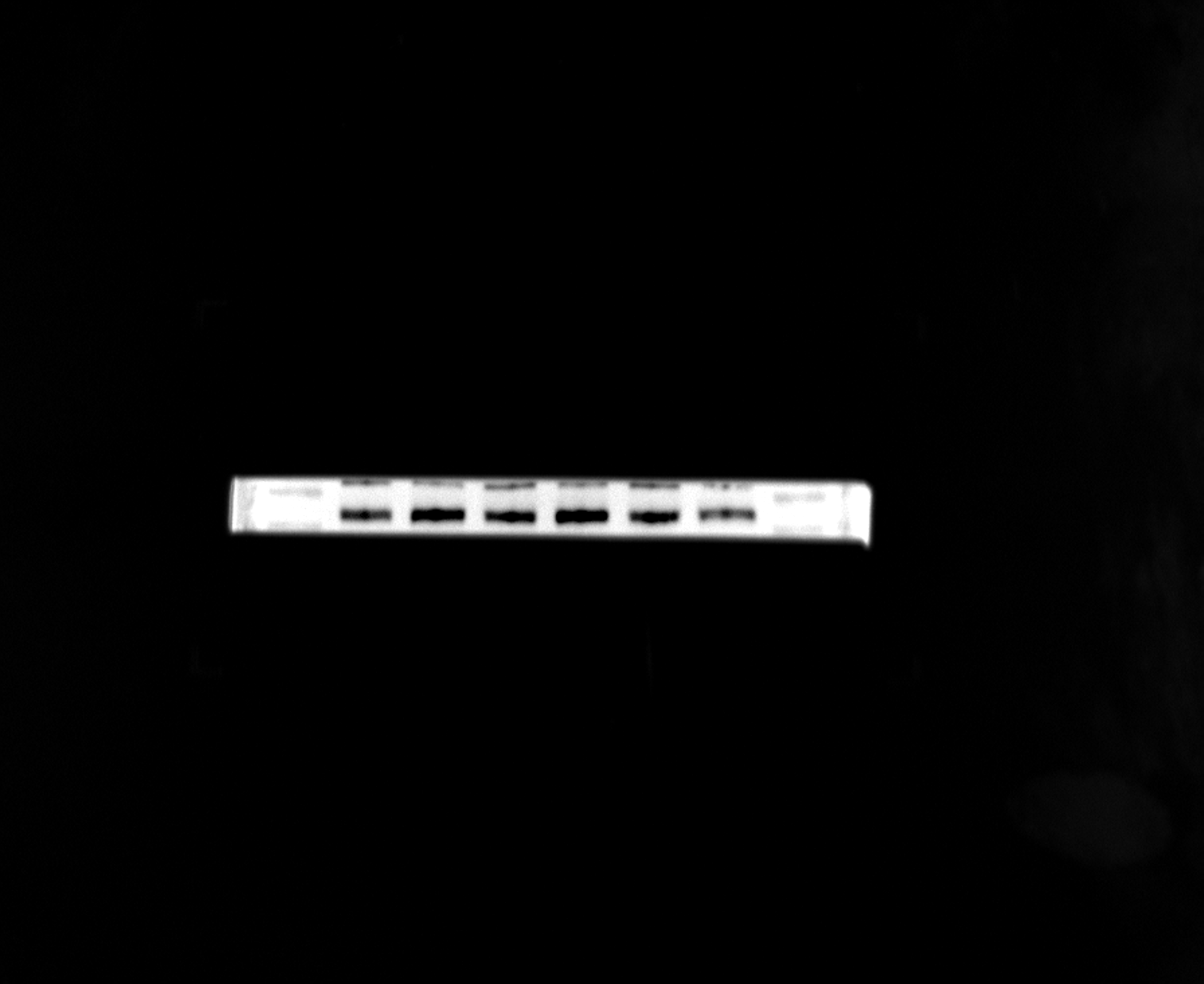

Supplement: Supplemental Information 9 [file peerj-13-20240-s009.zip › 1-GAPDH/1-cg-yg-Hat1.Tif]

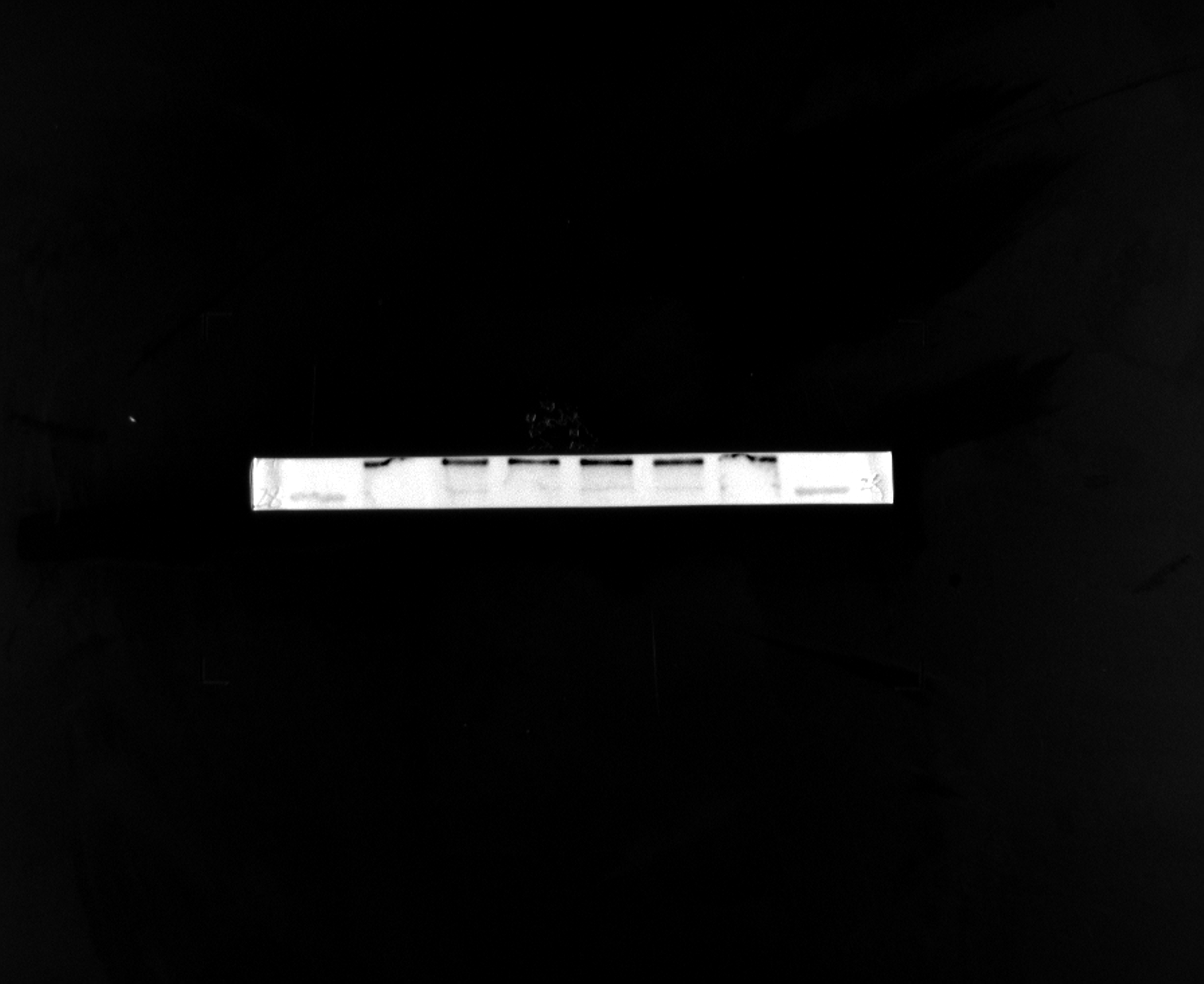

Supplement: Supplemental Information 9 [file peerj-13-20240-s009.zip › 1-GAPDH/2-cg-yg-GAP0DH.Tif]

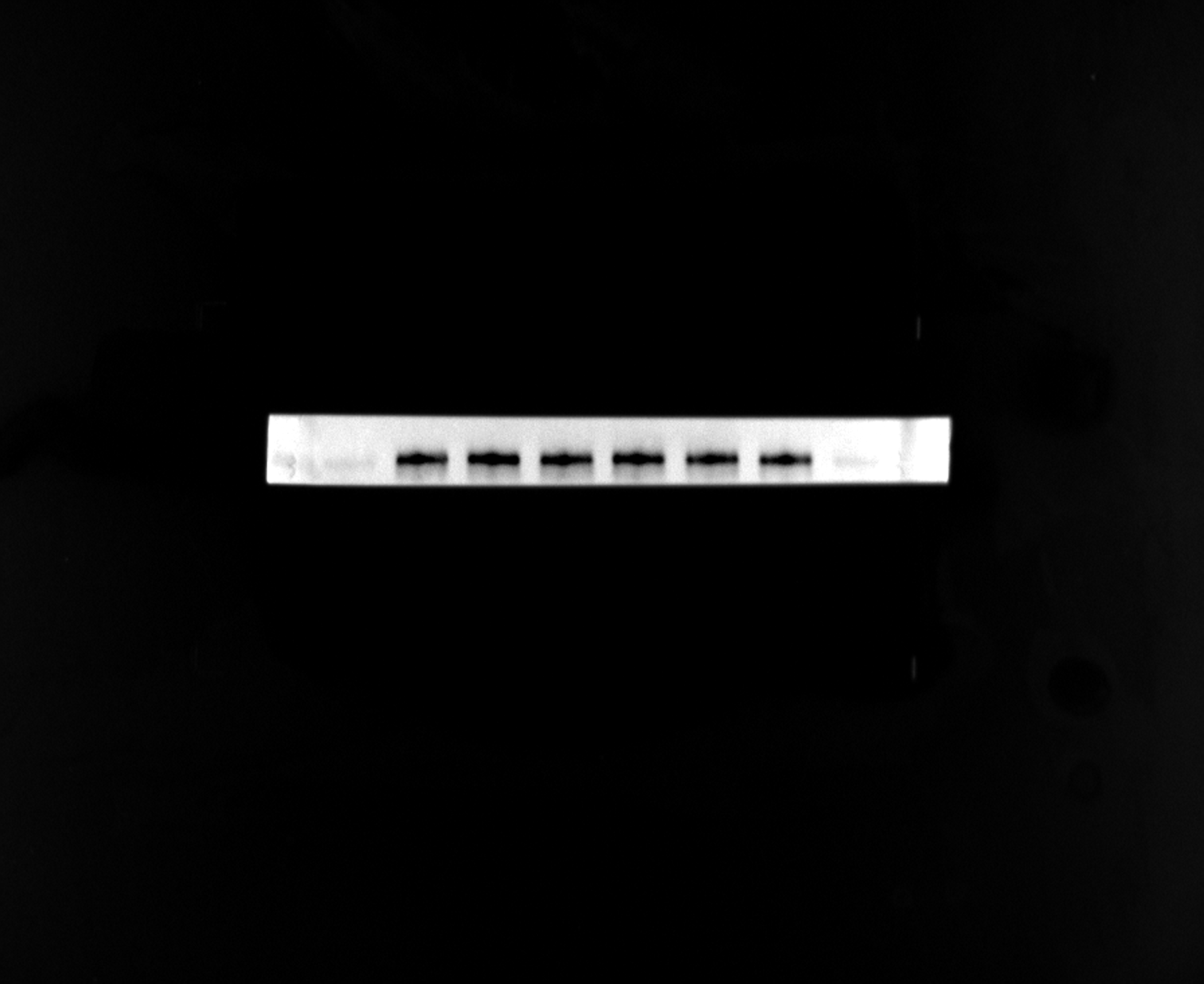

Supplement: Supplemental Information 9 [file peerj-13-20240-s009.zip › 1-GAPDH/3-cs-ys-GAP0DH.Tif]

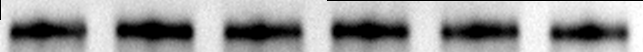

Supplement: Supplemental Information 9 [file peerj-13-20240-s009.zip › 1-GAPDH/4-cs-ys-GAP0DH.tif]

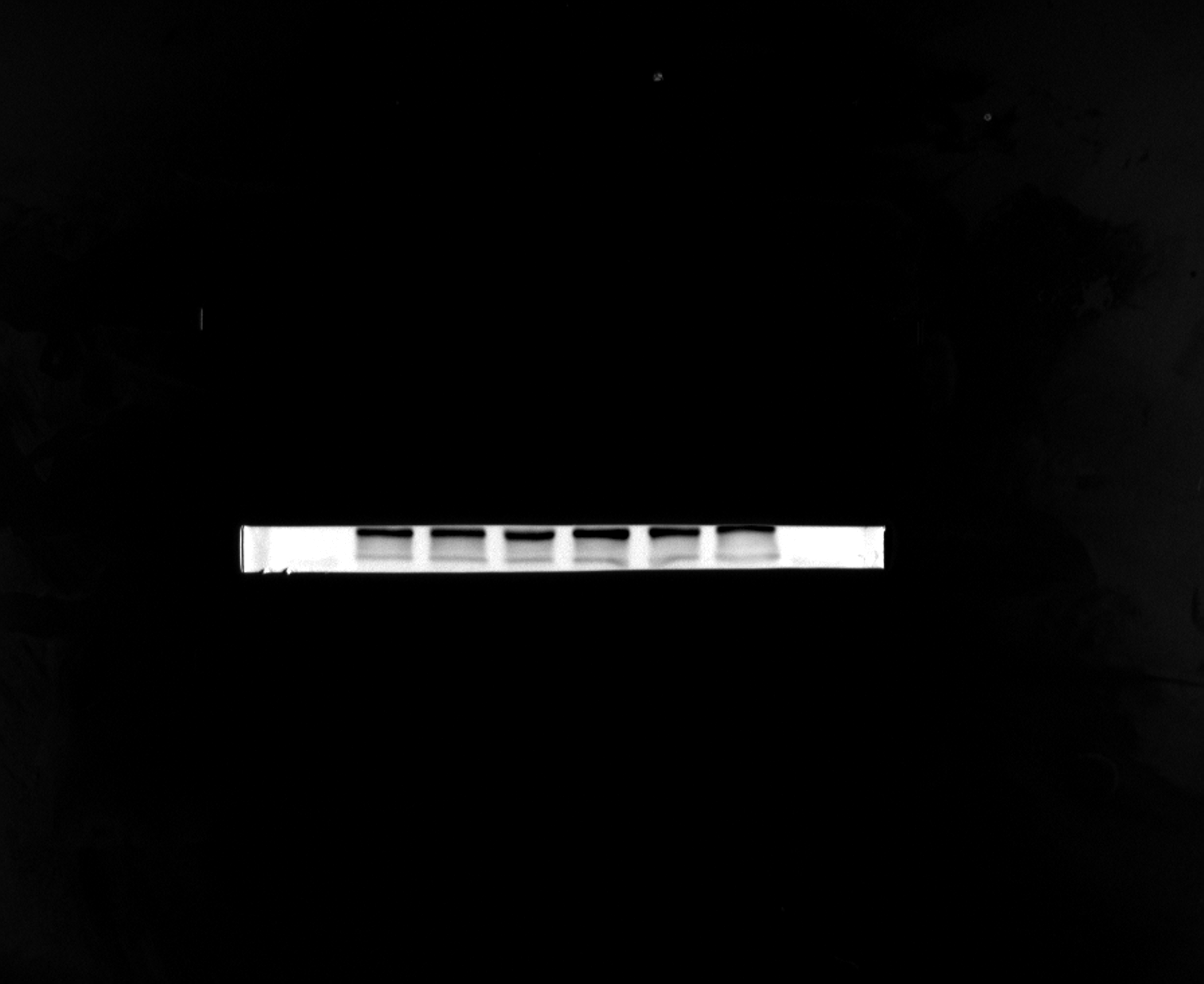

Supplement: Supplemental Information 9 [file peerj-13-20240-s009.zip › 1-GAPDH/5-cg-yg-GAP0DH.Tif]

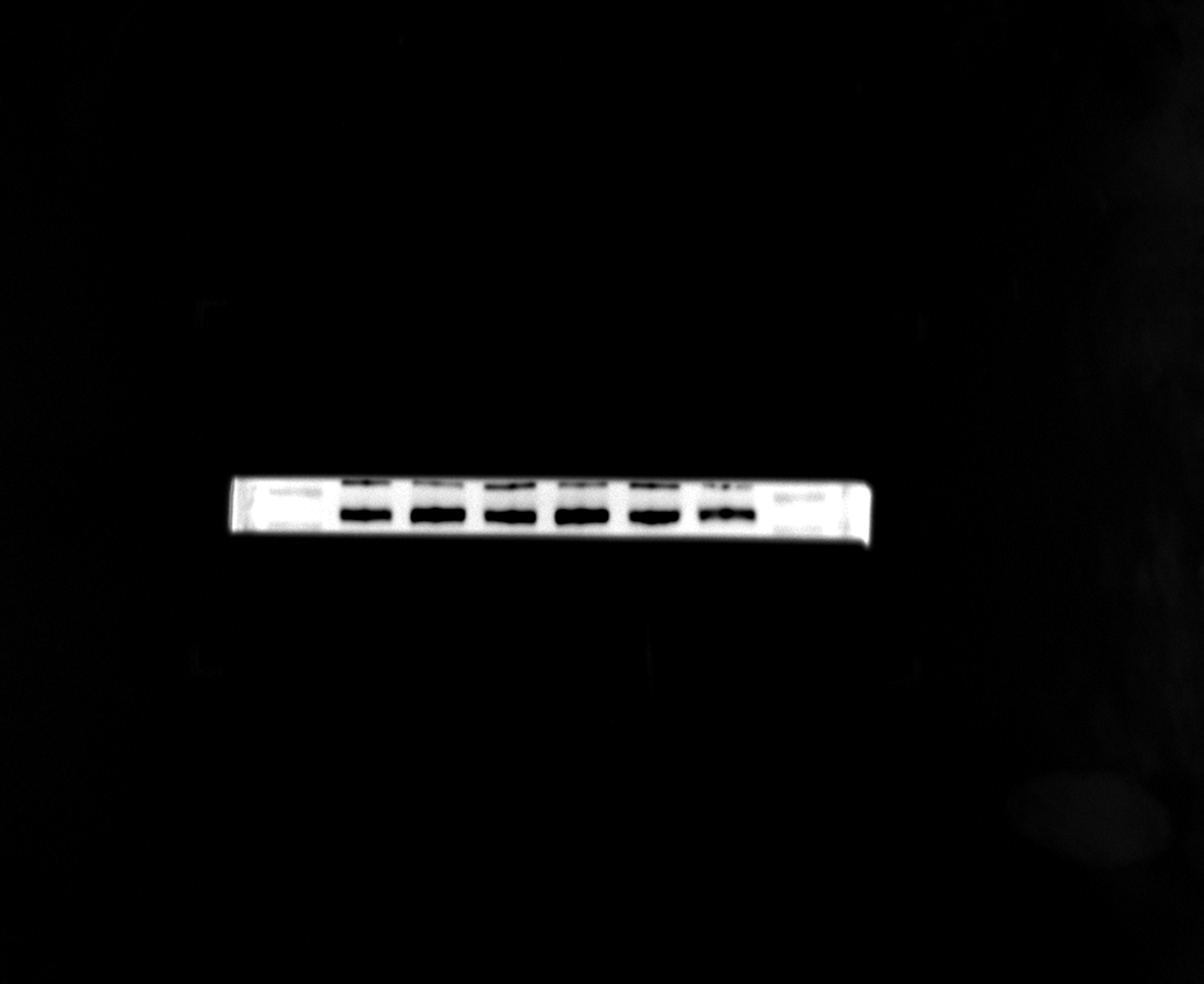

Supplement: Supplemental Information 9 [file peerj-13-20240-s009.zip › 2-HAT1/1-cg-yg.Tif]

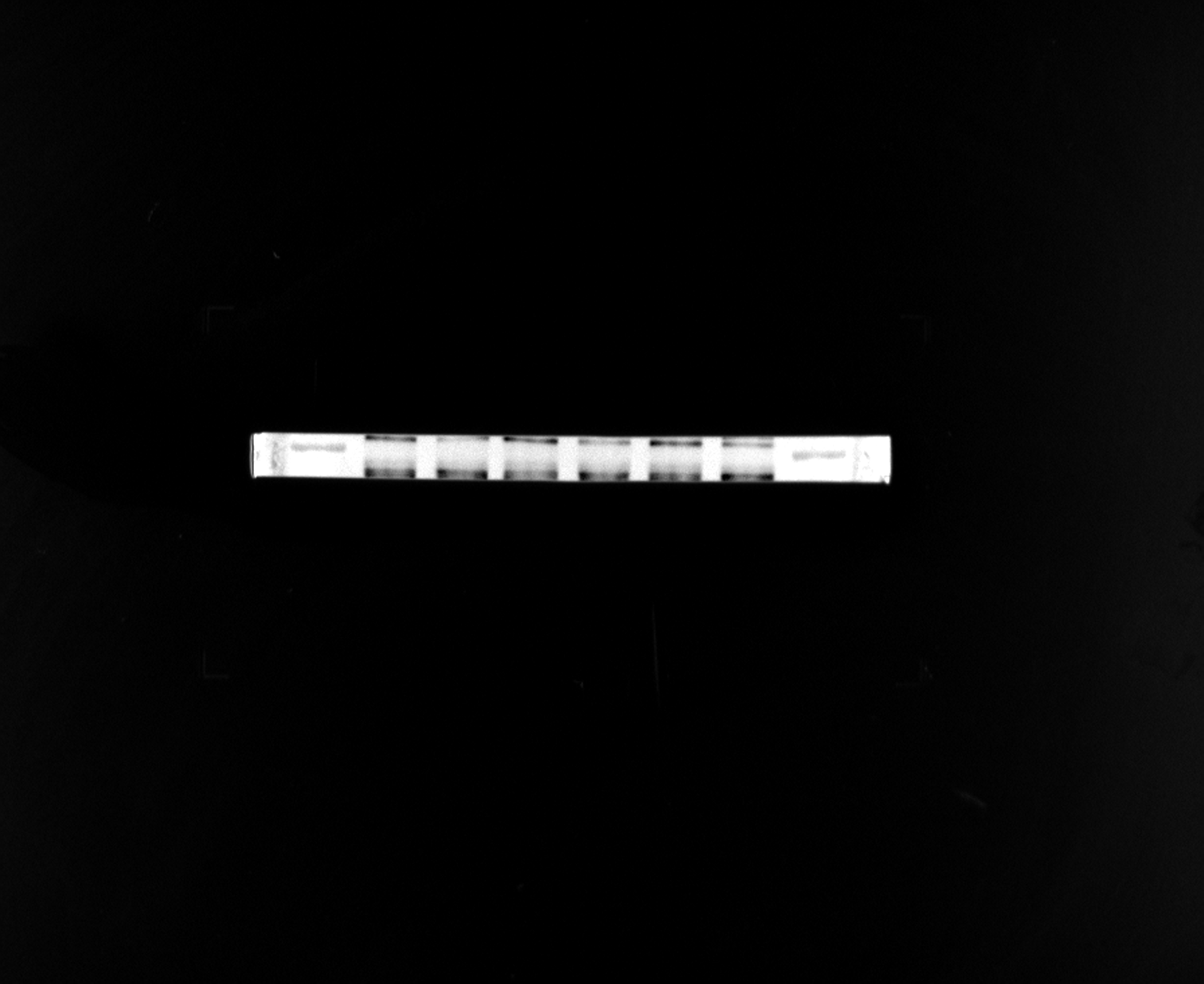

Supplement: Supplemental Information 9 [file peerj-13-20240-s009.zip › 2-HAT1/2-cg-yg.Tif]

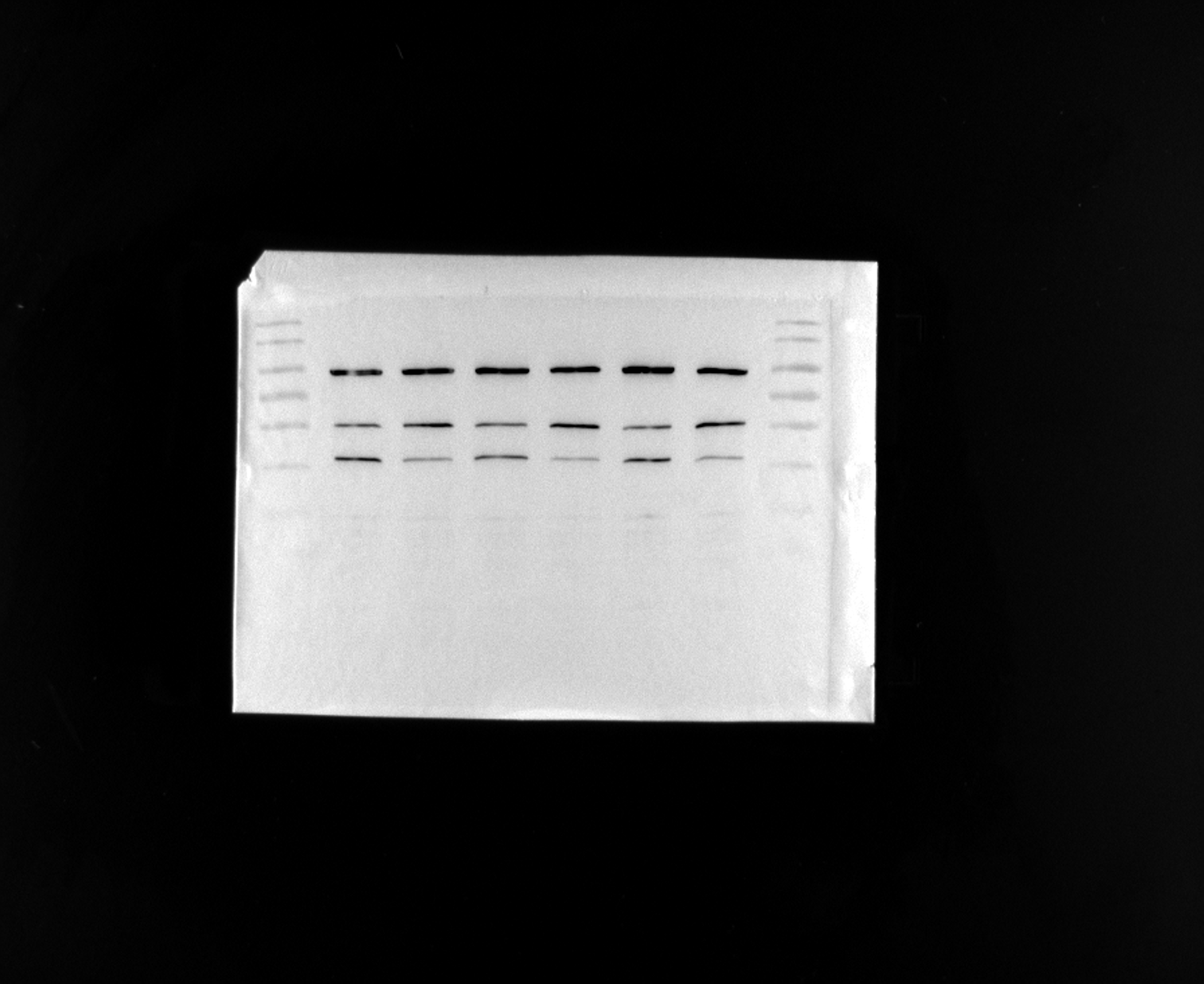

Supplement: Supplemental Information 9 [file peerj-13-20240-s009.zip › 2-HAT1/3-Hat1ys-cy-GAPDH.Tif]

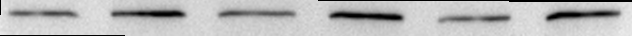

Supplement: Supplemental Information 9 [file peerj-13-20240-s009.zip › 2-HAT1/4-Hat1ys-cy-GAPDH.tif]

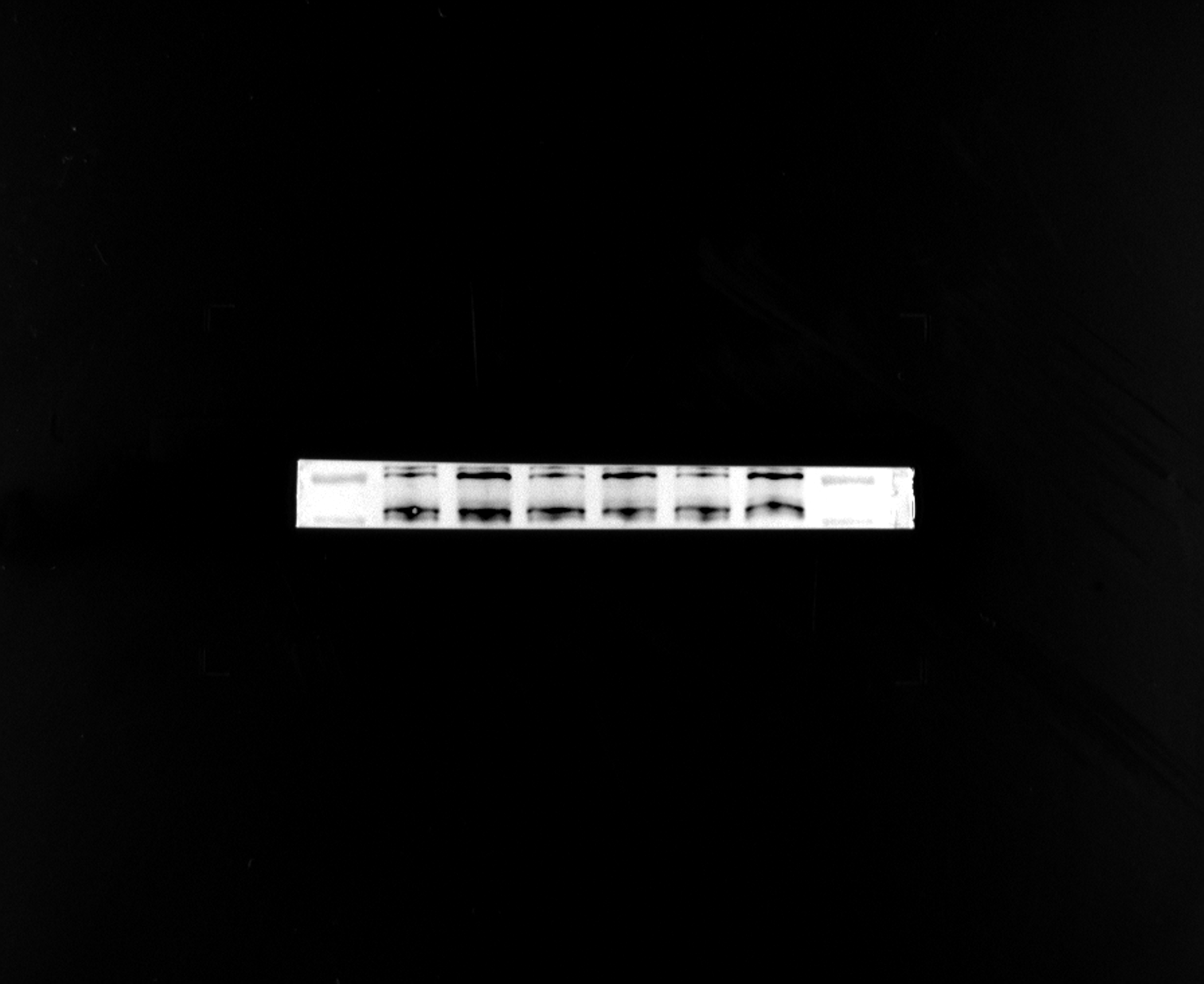

Supplement: Supplemental Information 9 [file peerj-13-20240-s009.zip › 2-HAT1/5-ys-cs-Hat1.Tif]

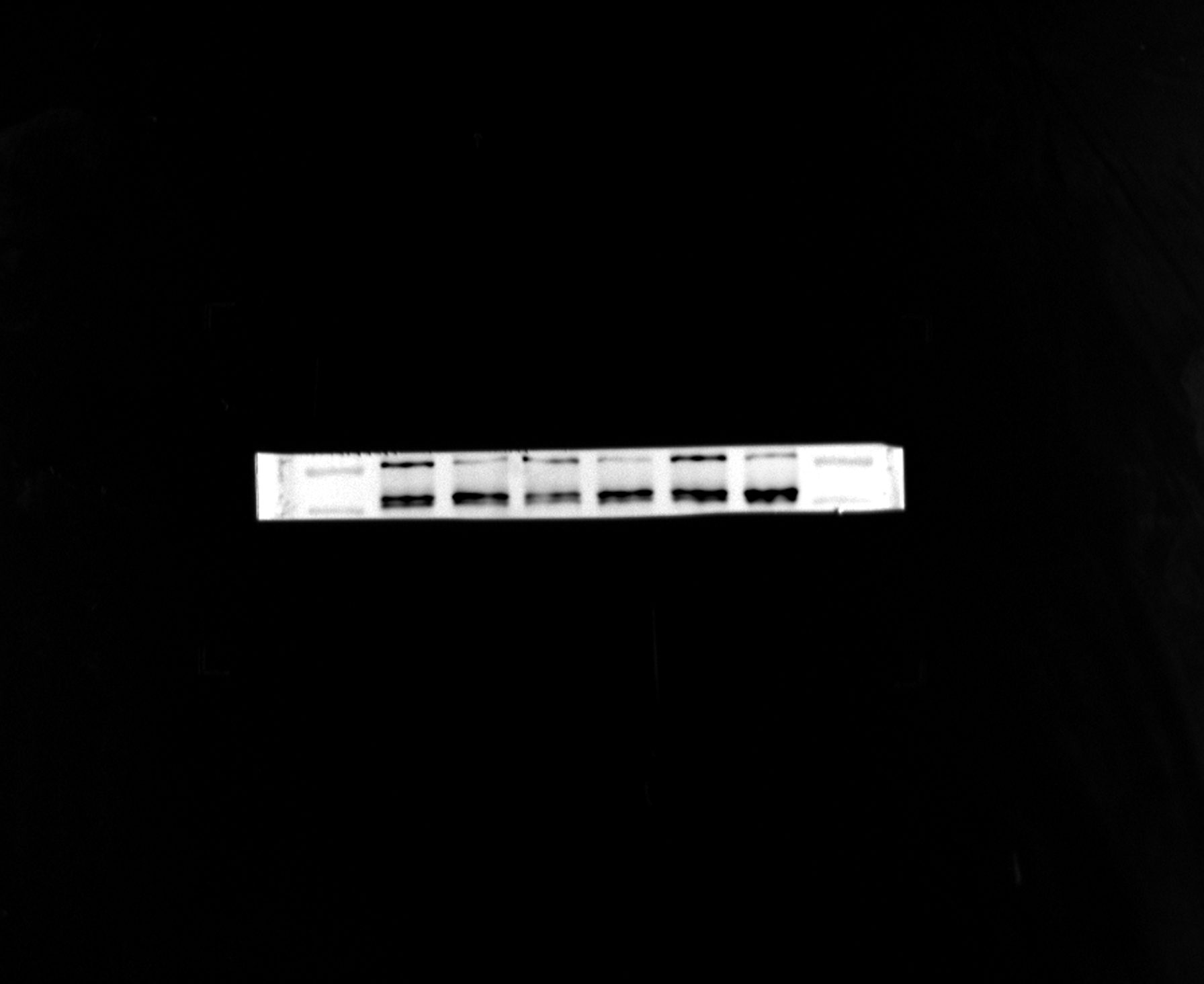

Supplement: Supplemental Information 9 [file peerj-13-20240-s009.zip › 2-HAT1/6-cg-yg-Hat1.Tif]

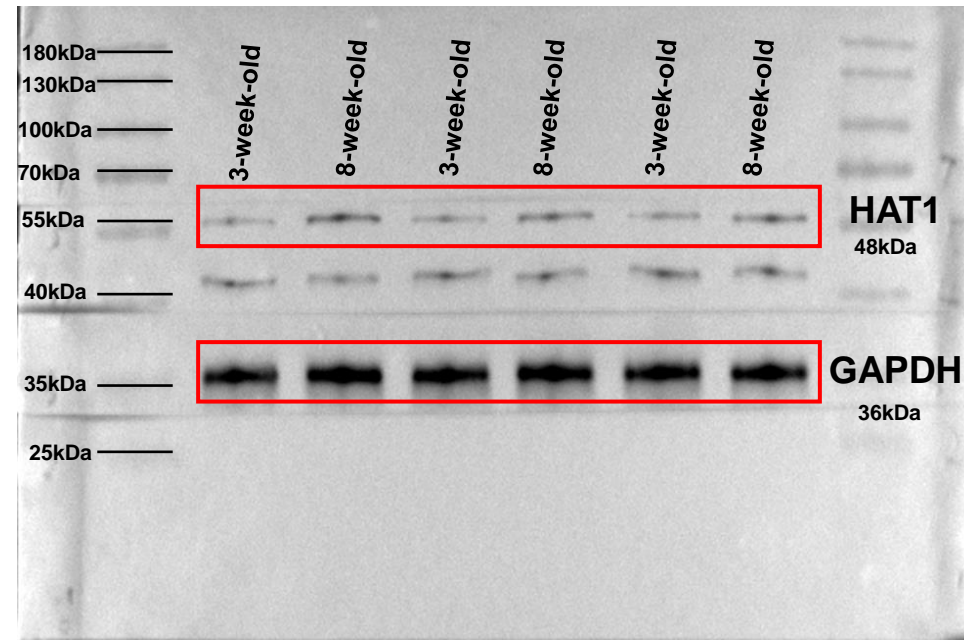

Supplement: Supplemental Information 9 [file peerj-13-20240-s009.zip › 3-integral film/WB-1.pdf]

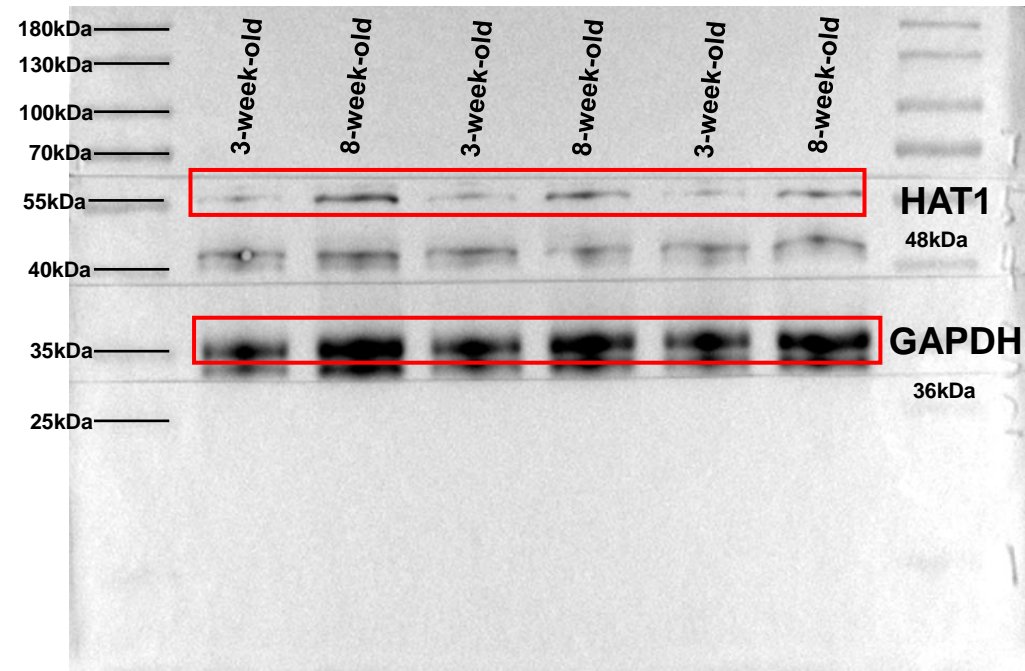

Supplement: Supplemental Information 9 [file peerj-13-20240-s009.zip › 3-integral film/WB-2.pdf]

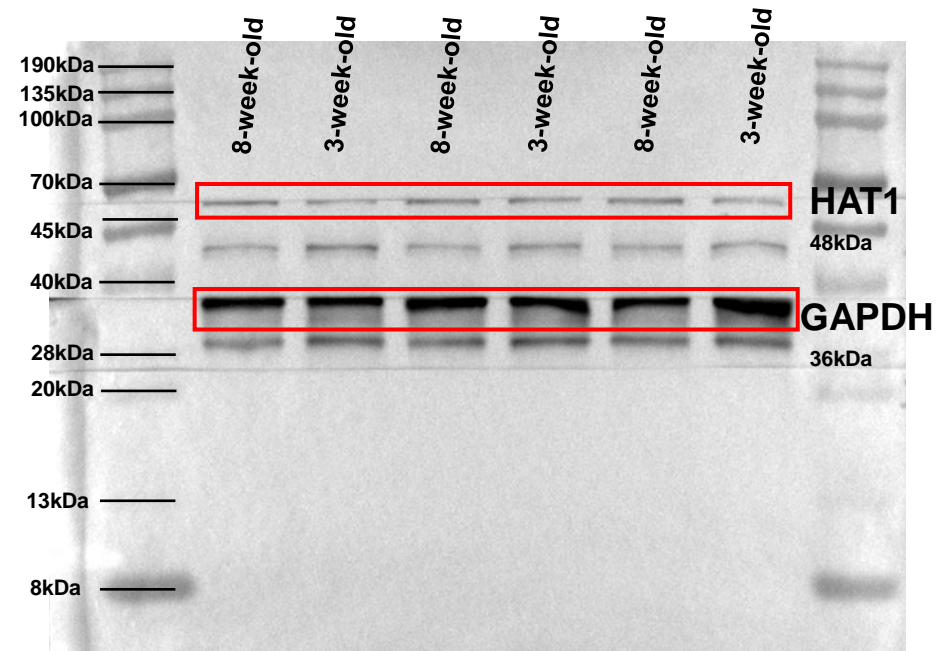

Supplement: Supplemental Information 9 [file peerj-13-20240-s009.zip › 3-integral film/WB-3.pdf]

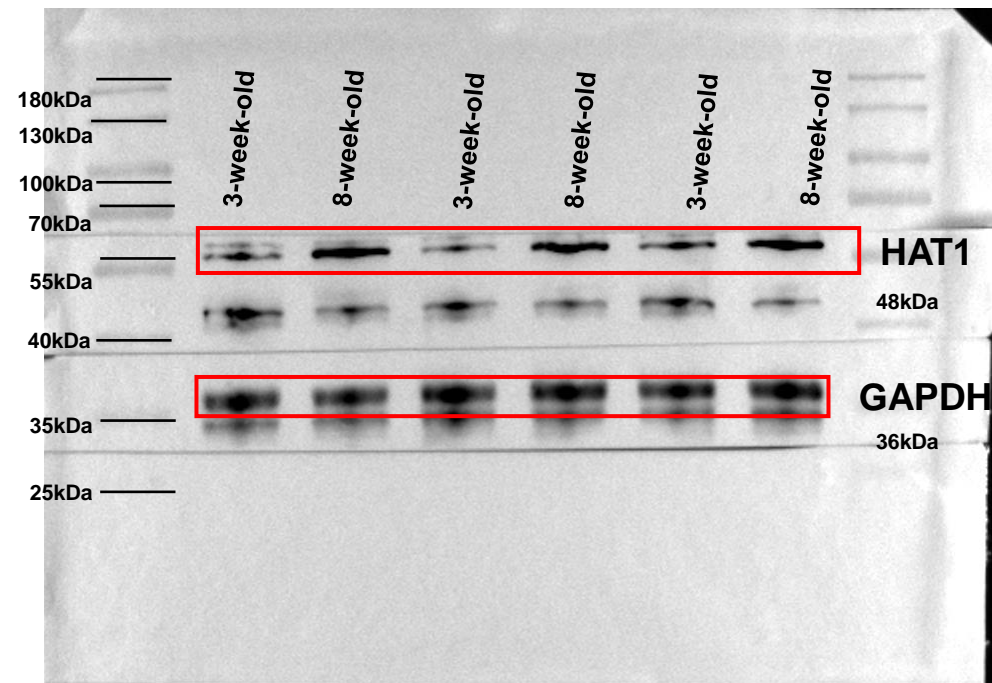

Supplement: Supplemental Information 9 [file peerj-13-20240-s009.zip › 3-integral film/WB-4 .pdf]

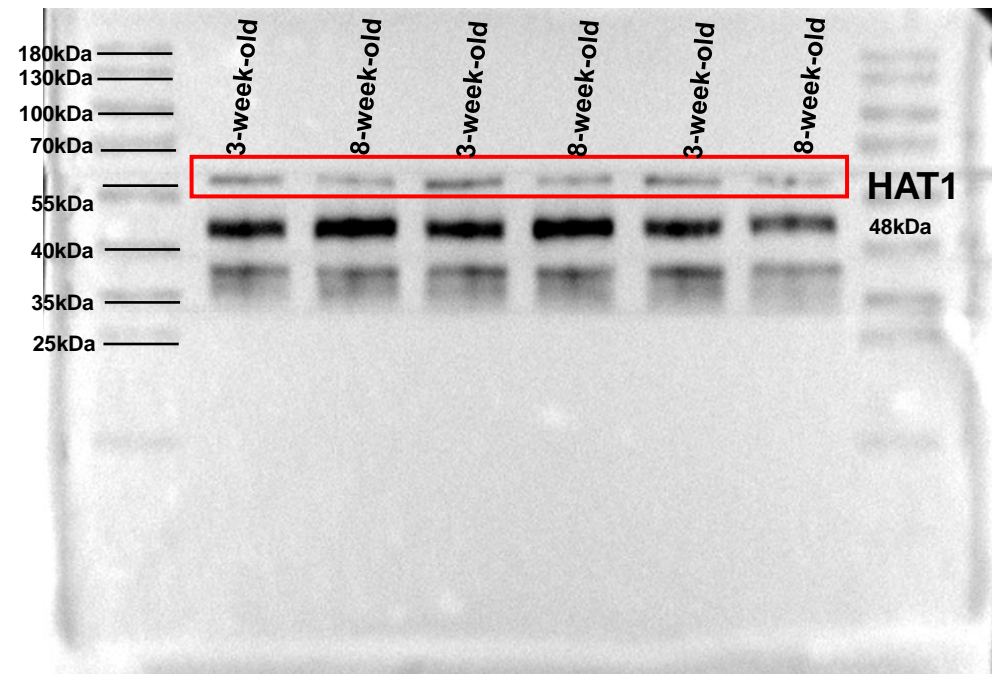

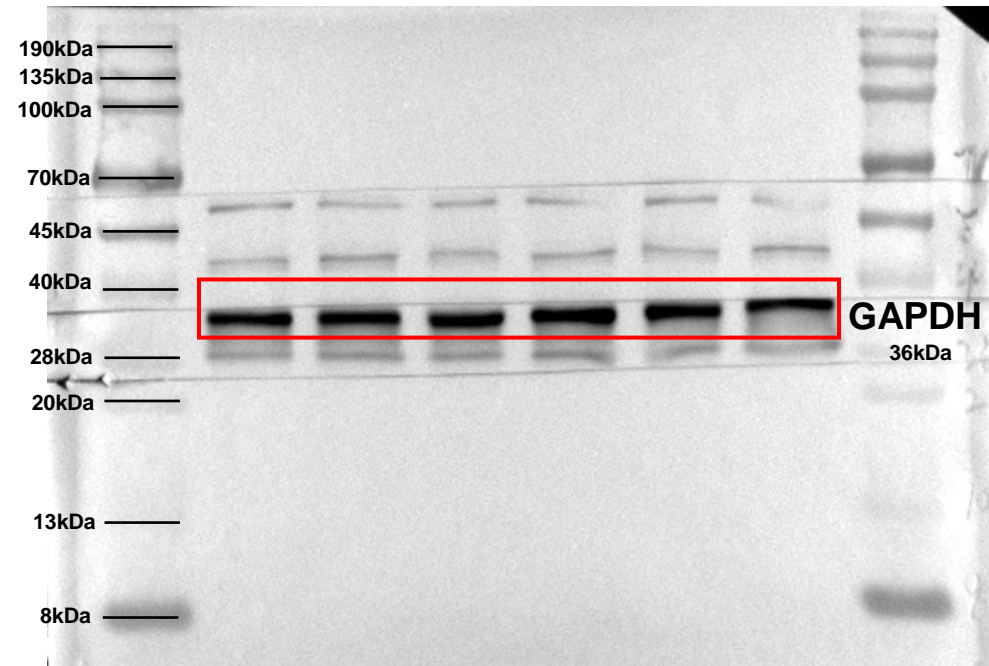

Supplement: Supplemental Information 9 [file peerj-13-20240-s009.zip › 3-integral film/WB-5.pdf]

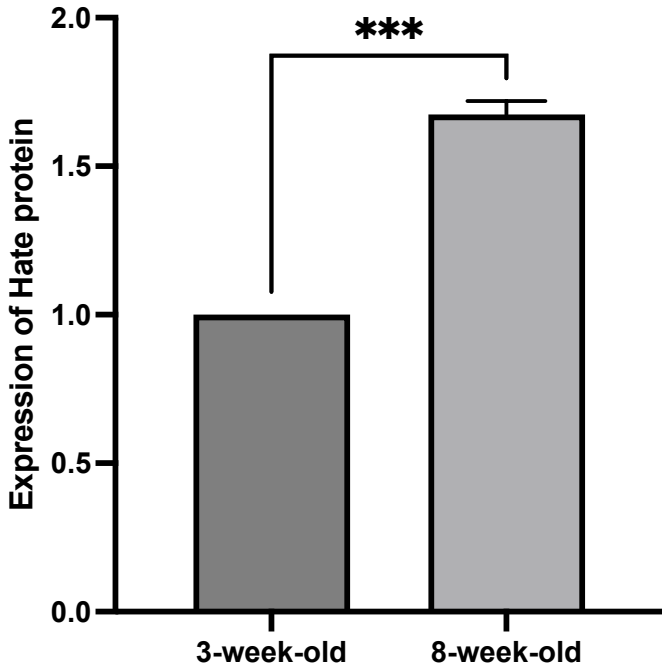

Supplement: Supplemental Information 9 [file peerj-13-20240-s009.zip › Expression of Hat1 protein.pdf]
