# Supplementary material for: Deciphering the role of Hat1 in spermatogenesis: Chromatin organization and beyond
Source: PeerJ. 2025 Nov 19;13:e20240. doi: 10.7717/peerj.20240 (PMC12640128; doi:10.7717/peerj.20240)
Supplement: Supplemental Information 11 [file peerj-13-20240-s011.doc]

**English Translation of Non-English Text in "Expression_of_Hat1_protein.xlsx"**

| **Non-English Original Text (Chinese)** | **English Translation (Academically Standard Expression)** | **Supplementary Note (Optional, to help the editor understand the context)** |
| --- | --- | --- |
| 内参 | Internal reference protein | Refers to GAPDH, the internal reference protein used for calibration in the experiment |
| 目的 | Target protein | Refers to Hat1, the target protein to be detected in the experiment |
